# Accelerated Mechanochemical Depolymerization of Poly(styrene) Due To Formation of a Cohesive State

**DOI:** 10.1002/cssc.70566

**Published:** 2026-04-05

**Authors:** Yuchen Chang, Aubrey M. Hepstall, Adrian H. Hergesell, Claire L. Seitzinger, Pawel Chmielniak, Ina Vollmer, Carsten Sievers

**Affiliations:** ^1^ School of Chemical and Biomolecular Engineering Georgia Institute of Technology Atlanta Georgia USA; ^2^ Inorganic Chemistry and Catalysis Institute for Sustainable and Circular Chemistry Utrecht University Utrecht Netherlands

**Keywords:** ball mill, depolymerization, polyolefin upcycling, solid‐state chemistry

## Abstract

Mechanochemical depolymerization in a ball mill can be used to convert poly(styrene) (PS) into monomeric styrene under milder conditions than thermal depolymerization. Continuous sampling of product flows shows that the rate of styrene formation increases significantly when PS powder is converted into a cohesive state, a viscous, continuous material phase that tends to coat the grinding spheres, once the temperature of the grinding surfaces approaches the PS glass transition temperature (~100°C). This enhancement is attributed to intensified mechanical shear stresses that generate favorable reaction environments for depropagation of chain‐end radicals in the bulk of the cohesive state. The faster depropagation steps also increase the selectivity of styrene relative to byproducts, such as methane, benzene, toluene, and ethylbenzene.

## Graphical abstract

A highly reactive, continuous state of poly(styrene) (PS) was discovered to aid its mechanochemical depolymerization, which is (a) more reactive than PS powder, (b) insensitive to effects of oxygen, (c) driven by shear rather than compressive stresses, and (d) sensitive to PS loading.

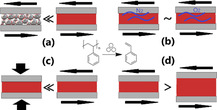

## 1 Introduction

Over the past two decades, mechanochemistry has steadily gained attention across many areas of chemistry as a facile way to perform solid‐state reactions [1, 2, 3]. In a typical laboratory setup, solid reactant powders are loaded into a jar together with grinding spheres. The jar is mounted onto an electrically powered ball mill that agitates the jar in a rapid periodic motion, causing collisions between the grinding bodies inside, which crush reactant particles in between [4]. Each collision subjects a small portion of reactant to transient high pressures, localized temperature spikes (hot spots), and other nonequilibrium conditions, thus providing the driving force for direct physical and chemical transformations of the reactant in the solid state [5].

It has long been observed for many inorganic mechanochemical reactions that product formation proceeds gradually at first before experiencing a sudden, dramatic increase in rate, often coinciding with a physical transition of the reactant material into an activated or critical state [6, 7, 8]. This behavior exhibits the signature of autocatalysis or positive feedback kinetics [9]. More recently, conceptually similar phenomena have been reported for organic mechanochemical batch reactions at the laboratory scale; the most well‐studied example is a Knoevenagel condensation [10, 11, 12] between vanillin and barbituric acid. Both reactants are solid powders at the start of milling, but once a threshold conversion has been attained, the dispersed powders become congealed into a continuous coating around the grinding sphere. In mechanochemistry literature, this continuous form of the reactants has been termed a ‘cohesive state’ [10]. The formation of cohesive states has resulted in sudden rate accelerations and rapidly completed reactions in many mechanochemical processes [13, 14, 15, 16, 17]. Likewise, a cohesive state accompanied by rate acceleration has been observed in the depolymerization reaction between poly(ethylene terephthalate) and sodium hydroxide [18, 19].

Although cohesive state kinetics have been observed across a diverse array of mechanochemical reactions involving more than one solid organic reactant, to the best of our knowledge there has been no reports of this phenomenon occurring in reactions involving just one solid‐state reactant. In this work, we report for the first time on the formation of what may be termed a rate‐enhancing cohesive state during the mechanochemical depolymerization reaction of poly(styrene) [20] (PS) conducted in a vibratory ball mill. In contrast to the slow intrinsic depolymerization kinetics of PS in the powder state at ambient mechanochemical conditions [21, 22], we show that PS depolymerizes at least one order of magnitude faster near its glass transition temperature of 100°C when it can be constituted as a viscous continuous substance (the cohesive state) and specifically under the action of shear forces. Polymers near their glass transition temperature are known to form a viscoelastic polymer state [23], which appears to cause cohesive behavior in a mechanochemical environment. The presence of oxygen—which enhances depolymerization in the powder state—does not significantly affect PS depolymerization in the cohesive state. These insights have practical implications for the potential use of mechanochemistry to achieve solid‐state depolymerization of presently difficult‐to‐recycle olefinic plastics.

## 2 Experimental Section

### 2.1 Materials

PS pellets with weight‐average molecular weight (*M*_w_) = 192,000 g/mol (reported by manufacturer) were purchased from Sigma–Aldrich. Pellets were crushed for 8 min using a Retsch PM200 planetary mill in 50 g batches with five 20 mm diameter steel spheres inside a 125 mL steel grinding jar. The crushed PS grains retained between 16‐ and 60‐mesh sieves (250–1180 μm in size) were used in depolymerization experiments. Pre‐grinding of PS did not significantly affect the molecular weight (MW) distribution of the material [21]. Reagents used in preparation of samples for characterization, such as methanol (≥99.9%) and decane (≥99%), were purchased from Sigma–Aldrich and used without further purification. Ultra‐zero‐grade air and ultra‐high‐purity grade N_2_ were purchased from Airgas.

### 2.2 Ball Milling Experiments

Experiments were conducted on two different setups using the same reactor design and operating conditions, and different instrumental setups for product analysis termed discrete sampling and continuous sampling, described in separate paragraphs below. Depolymerization experiments were conducted in a modified tungsten carbide‐lined grinding jar manufactured by Retsch and tungsten carbide grinding spheres. The jar had a pill‐shaped internal volume of 25 mL and two openings on the cylindrical face to allow connections to gas lines via Swagelok fittings welded to the jar. Grinding spheres with masses ranging from 7.8 to 53 g were used in experiments. Reactor loadings were 1.0–3.0 g of pre‐ground PS. The reactor was connected to 3.18 mm poly(propylene) tubing using Swagelok unions, and gas (N_2_ or air) flowing at 60 mL/min was maintained during milling using a mass flow controller upstream from the jar inlet line. All experiments were performed at 30 Hz operating frequency on a Retsch‐manufactured shaker mill. The atmosphere surrounding the ball mill was either stagnant air to allow temperature development in the reactor to equilibrate naturally with the surroundings during milling or rapidly flowing air to achieve passive cooling and maintain the reactor exterior surface near ambient temperature.

*Discrete sampling* experiments were conducted on a Retsch MM400 shaker mill. Downstream from the outlet line of the reactor a gas dispersion tube was used to bubble effluent gas from the reactor into a methanol (MeOH) solvent trap (containing 10 mg of decane as an internal standard) to collect volatile products from the reaction. Traps were switched out for new ones at time intervals of 10 or 30 min to prevent styrene buildup. Analysis of the trap samples was performed on a Varian‐Bruker 450‐GC equipped with a Supelco SPB−1 fused silica capillary column and flame‐ionization detector (FID)—the latter was augmented by a Polyarc quantitative carbon analyzer manufactured by Activated Research Company. The carrier gas was helium at 2 mL/min. The yield $Y_{j}$ (product mass per unit mass of PS) of each product compound j was calculated using its chromatogram peak integration area $I_{j}$, molecular weight $MW_{j}$ and carbon number $N_{j}$ and the same quantities ($I_{\mathrm{dec}}$, $MW_{\mathrm{dec}}=142.3$ g/mol, $N_{\mathrm{dec}}=10$) for decane, normalized by the initial mass of PS $m_{\mathrm{PS},0}$ in the reactor, according to

(1) $$Y_{j}=\frac{I_{j}}{I_{\mathrm{dec}}}\cdot \frac{MW_{j}}{MW_{\mathrm{dec}}}\cdot \frac{N_{\mathrm{dec}}}{N_{j}}\cdot \frac{m_{\mathrm{dec}}}{m_{\mathrm{PS},0}}$$

as was described in previous work [21]. *Continuous sampling* experiments were conducted on a Retsch MM500 Vario mixer mill. Volatile products in the effluent gas stream were sampled downstream by an in‐line gas chromatograph (GC, manufactured by Global Analyser Solutions) equipped with four parallel column channels for the quantification of N_2_, C_1–3_, C_4–7_, and C_5–10_ products, respectively. N_2_ was quantified using a thermal conductivity detector (TCD), while hydrocarbon products were quantified using flame ionization detectors (FIDs). Sampling from the effluent stream occurred once every 1.05 min on the N_2_ channel, 2.1 min on the C_1–3_ channel and 4.2 min on the other two hydrocarbon channels. The effluent gas flow rate was maintained at a constant $F_{0}=60$ mL/min, which was used as an internal standard to counter changes in total flow $F_{\mathrm{tot},i}$ due to the formation of depolymerization products. For the ith injection, $F_{\mathrm{tot},i}$ was calculated by comparing the N_2_ TCD peak area $A_{N_{2},i}$ of this injection with the average peak area from three N_2_ blank injections ($A_{N_{2},b1}$, $A_{N_{2},b2}$, $A_{N_{2},b3}$) preceding the start of milling. Molar flow $F_{P,i}$ of each hydrocarbon product P detected in the ith injection is then calculated from its FID peak area $A_{P,i}$ according to

(2) $$F_{P,i}=F_{0}\left(\frac{A_{N_{2},b1}+A_{N_{2},b2}+A_{N_{2},b3}}{3A_{N_{2},i}}\right)\cdot \frac{1}{y_{N_{2},0}}\cdot \frac{A_{P,i}}{K_{P}}$$

where $y_{N_{2},0}$ is the N_2_ molar fraction in pure purge gas, and $K_{P}$ is a calibration factor determined by calibration of the FIDs with a mixture of methane, ethane, propane, butane, hexane, and heptane. A continuous feedback thermocouple was fastened to the exterior surface of the reactor for real‐time temperature monitoring. These procedures have been adapted from previous work [24].

### 2.3 Sound Recording and Analyses

Audio recordings of experiments were collected using a FIFine Technology USB microphone interfaced with Audacity (versions 3.7.3‐4). Audio was tracked in mono at a recording level of 20% (chosen as reference point of maximum intensity in a given experiment at 0 dB) and a sampling rate of 176,400 Hz (in 32‐bit format). The microphone was placed in front of the shaker housing the reactor jar with the diaphragm facing upward. Recordings were exported from Audacity as .wav files and processed in Python using the librosa package.

## 3 Results and Discussion

This section is structured into two parts. First, we describe how PS reproducibly transitions suddenly from a granular powder to a viscous, cohesive state of PS under suitable mechanochemical conditions. This occurs when the temperature of the grinding media approaches the PS glass transition temperature (~100°C), and therefore, the resulting soft phase that coats the grinding spheres is formed thermally. Second, through reactivity data (see Table 1), we show that depolymerization is enhanced by an order of magnitude in the cohesive state, which can be rationalized from a thermodynamics perspective by elevated local temperature in the cohesive state reaction microenvironment relative to PS powder. Kinetically, however, it is shear rather than compressive stresses experienced by PS during grinding collisions that are largely responsible for the production of styrene and byproducts. The rate of monomer production becomes insensitive to O_2_ in the cohesive state, in contrast to the enhancing effect of O_2_ in depolymerization kinetics of PS powder [21].

**TABLE 1:** Discrete sampling experiments. All experiments were conducted at 30 Hz milling frequency in WC grinding tools with a purge gas flow rate of 60 mL/min. Initial rates refer to styrene production.

Exp. #	Number × size (mm) of spheres	Initial mass (g) of PS	Gas phase	Cooling	Time^a^ (min) to cohesive state	Initial rate^b^ (powder) (mg/g PS/min)	Initial rate^c^ (cohesive) (mg/g PS/min)
1	8 × 10	1.0	N_2_	No	16	0.14^d^	1.67^d^
2	8 × 10	1.5	N_2_	No	28	0.06	1.10
3	8 × 10	2.0	N_2_	No	32	0.02	1.03
4	8 × 10	3.0	N_2_	No	n.a.	~0	n.a.
5	8 × 10	1.0	Air	No	17	0.30	1.61
6	8 × 10	1.0	N_2_	Yes	n.a.	0.14	n.a.
7	8 × 10	1.0	Air	Yes	n.a.	0.36	n.a.
8	1 × 19	1.0	N_2_	No	6	~0	n.a.
9	2 × 15	1.0	N_2_	No	12	0.14	0.84
10	4 × 12	1.0	N_2_	No	12	0.23	2.65

a Time from the start of milling to the transition in the sound spectrum that indicated the formation of the cohesive state (see Figure S7 for illustration of how this time interval was identified from audio recording).

b Calculated using the first pair of data points (cumulative yield over 10‐min interval) recorded before the time interval during which the cohesive state was formed (see Figure 3a,b).

c Calculated (where applicable) using the first three data points recorded after the time interval in which the cohesive state was formed (see Figure 3a,b).

d Uncertainty in initial rates is generally ±0.03 mg/g PS/min for powder state milling and ±0.17 mg/g PS/min for cohesive state milling, from the average of three experiments.

### 3.1 Formation and Characteristics of a Cohesive State in PS

PS powder was milled in a shaker mill using a 25 mL tungsten carbide (WC) jar with high‐density WC spheres and an atmosphere of air or N_2_. After an incubation period of 10–30 min, the powder underwent a physical transformation by agglomeration into a viscous continuous layer that typically coated the WC spheres and sometimes over the reactor wall. This substance is hereby called the cohesive layer or cohesive state. The incubation time depends primarily on the PS loading inside the reactor, the configuration of grinding spheres used in milling, and the exterior environment of the reactor, as will be discussed in the next subsection.

**FIGURE 1:**
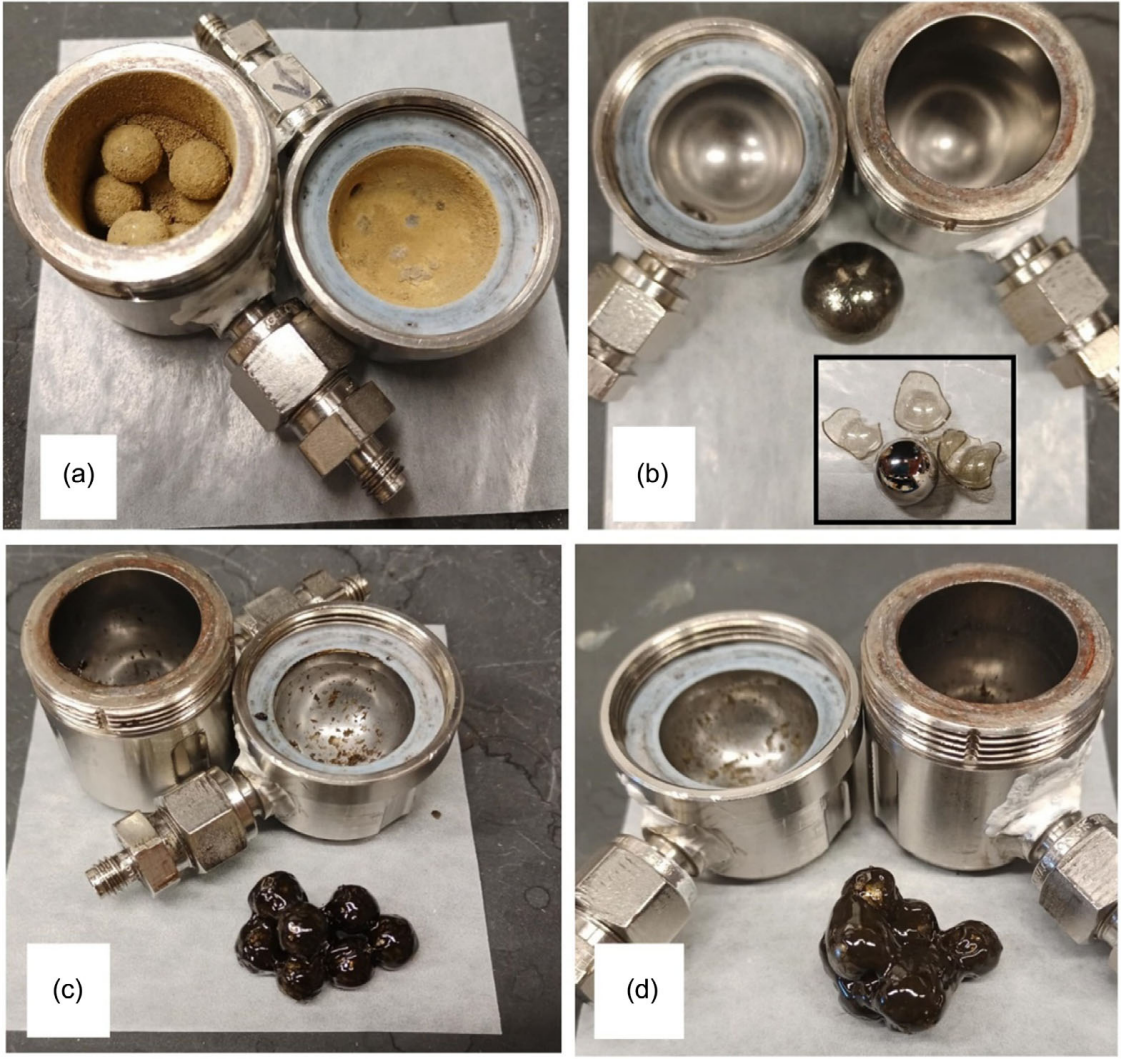
Appearances of PS as powder and cohesive states after grinding using WC jar and spheres; (a) powder (Table 1, Exp. 6), and cohesive states formed using (b) one 19 mm sphere and 1 g PS (Table 1, Exp 8) with insert showing the cohesive layer broken off from the sphere as rigid pieces after cooling to ambient temperature, (c) eight 10 mm spheres and 1 g PS (Table 1, Exp. 1), (d) eight 10 mm spheres and 2 g PS (Table 1, Exp. 3).

With an appropriate loading of PS powder and suitable operating conditions (see Table 1) that allow high‐energy milling, transformation of PS from powder to a cohesive material was achieved using one single large (19 mm) sphere or several smaller spheres (for example, two 15 mm, four 12 mm, or eight 10 mm spheres). With one single sphere, the congealed PS formed a nearly smooth and uniform coating over the entire surface of the sphere, leaving no residue on the jar surface. When more than one grinding sphere was present in the reactor, the viscous PS congealed all the spheres together into a single mass, and the distribution of PS over the grinding sphere surfaces was not uniform; some specks of residue were also observed on the jar surface. The photographs in Figure 1 depict examples of the physical appearance of the cohesive layer formed using one 19 mm sphere (b) or eight 10 mm spheres (c–d), contrasted with the appearance of PS during grinding in powder form (a). As expected, 2 g of PS (d) formed a visibly thicker layer compared to 1 g (c). Coloration of the PS in Figure 1a, c,d can be attributed in part to fine WC particles from attrition to the WC spheres and jar, and also partly to small quantities of coke species formed during depolymerization reactions [22].

In all experiments, milling commenced with the sphere(s) and jar at ambient temperature. During ball milling, the temperature of the grinding surfaces (spheres and jar interior wall) increased steadily and—in the absence of deliberate convective cooling by an air stream directed at the exterior of the jar—asymptotically approached a steady state value at least 50°C above ambient temperature, which occurred within 1 h of uninterrupted milling [25]. This behavior is well documented with shaker mills [26, 27] and is due to the gradual accumulation of heat dissipated from collisions inside the jar. Immediately after milling was stopped, the jar was opened, and a thermocouple was used to take temperature readings of the grinding surfaces—this temperature was found to be 100±10°C, which coincides with the glass transition range of PS (90–106°C) [28, 29]. The measured temperature of the cohesive layer itself exceeded 100°C, and the soft and rubbery material exhibited plastic deformation similarly to bread dough or putty when poked.

For the same milling time, cohesive state formation was suppressed by maintaining steady air flow around the reactor as a heat exchanger to maintain the reactor exterior surface at ambient temperature. From this observation, it is evident that the formation of the cohesive state of PS was caused when the heat released by impacts during milling increased the temperature of PS to a critical point. Once the surface temperature was close to the glass transition range of the PS, the glassy particles became sufficiently softened and congealed into a continuous layer—the cohesive state. In the cohesive state, most of the PS ended up coating over the spheres because their surfaces tend to be the hottest inside the reactor environment [30] and therefore the earliest to attain the glass transition range of PS. Conversely, heating the reactor externally using a heat gun led to the cohesive layer to develop on the reactor wall instead of the spheres (see Figure S1j, Supporting Information).

Upon cooling, the viscous PS hardened into a rigid and brittle coating holding the spheres together in a rigid mass as those depicted in the photographs of Figure 1b‐d. This hardened PS easily reverted back to the powder form depicted in Figure 1a by gentle mechanical agitation at a milling frequency of 10 Hz once returned to the mill together with grinding spheres. Upon resumption of milling, the reconstituted powder could be brought into the cohesive state again (having the same visual appearance) within an incubation time 2–5 min less than the initial duration required to bring fresh PS powder into the cohesive state. For the unmilled PS which has a number average MW (*M*_*n*_) of ~86,000 g/mol (Table S1), the glass transition temperature is about 104°C (which asymptotically approaches 106°C with increasing MW) [31]. Meanwhile, MW degradation of PS by shaker milling from any starting *M*_*n*_ is known to occur predominantly in the initial minutes of grinding [21] and asymptotically approach the characteristic limiting *M*_*n*_ = 10,000 g/mol at long milling times [32], corresponding to a glass transition temperature of only around 95°C [31]. During the time required to bring fresh PS powder to the cohesive state, the MW degradation will reduce the glass transition temperature of the material by several degrees. Upon resumption of milling, less time is required for the reactor interior to heat from ambient temperature to the lower glass transition temperature of partially degraded powder than to the asymptotic 104°C that characterizes the unmilled powder. This explains why the cohesive state forms in a shorter incubation time for PS powder that has already been milled to a lower *M*_*n*_ than fresh powder starting from a higher *M*_*n*_.

**FIGURE 2:**
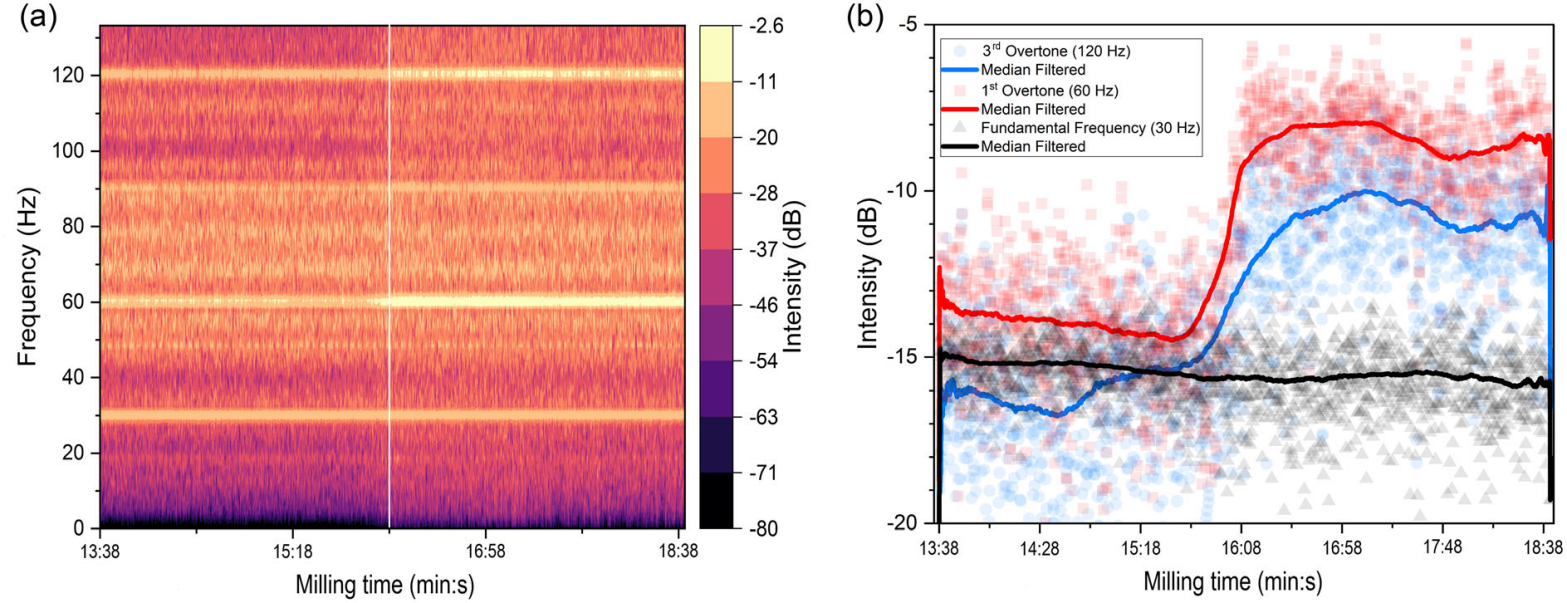
(a) Audio spectrogram and (b) intensity analysis for characteristic frequencies during a 5‐minute time interval encompassing the physical transition from powder to cohesive state with eight 10 mm spheres and 1 g PS (conditions corresponding to Exp. 1 in Table 1). Time scale refers to time elapsed since start of milling. White line in (a) denotes time of transition in the actual experiment (16 min from the start of milling, see Figure S7).

Physical transition of the PS from powder to a fully developed cohesive layer occurred quickly and was accompanied by an audible increase in the intensity of collision sounds emanating from inside the mill (Figure S6, Supporting Information). Figure 2 depicts the time‐resolved intensity spectra of a 5‐min audio segment encompassing the cohesive layer formation clipped from a longer recording (Figure S7) of an experiment (Exp. 1, Table 1) where the transition to cohesive state occurred at around 16 min into milling (corresponding to position of the white line in Figure 2a). During the entire milling time, the fundamental frequency (30 Hz) remained constant (see contrast with background spectrograms in Figure S6a,b, Supporting Information). An abrupt increase in intensity was observed within a very short time span of less than 15 s. During this time, the overtones of the fundamental milling frequency (particularly 60 and 120 Hz) increased in intensity from –15 dB to –(10–9) dB relative to the reference level (0 dB) (Figure 2b). The intensification of these harmonic peaks is consistent with a transition in the mechanical behavior of the milling media from many semi‐independent moving bodies to a single mass colliding as one unit with the reactor wall. The audio spectra for milling with four 12 mm spheres exhibited the same essential characteristics as the plots in Figure 2 (compare Figure S6c, d,k in Supporting Information), whereas with a single 19 mm sphere or two 15 mm spheres, the contrast in acoustic intensity before and after the physical transition was less pronounced (see Figure S6i,j) due to these configurations of spheres behaving more like a single body during milling regardless of the cohesive state. An acoustic signature of this sort is consistent with observations from recent operando methodologies that used in situ audio monitoring to detect phase transitions and bead‐motion changes in mechanochemical systems [33].

Formation of the cohesive state in PS was not specifically reliant on the use of WC grinding jars and spheres. Rather, the steady‐state internal temperature of the WC reactor situated in stagnant air attained the glass transition temperature range of PS from a variety of milling configurations (number and sizes of spheres), so the same outcome could be obtained using one large sphere or several smaller ones. On the other hand, a steel reactor surrounded by stagnant air could only reach a steady state internal temperature of 100°C from grinding PS with one large (≥20 mm) steel sphere (see Figure S1b, Supporting Information). Formation of the cohesive state was indeed observed by milling PS in this configuration, keeping all other operating parameters identical to the WC milling experiments. Conversely, collisions of many small 10 mm steel spheres were insufficient to accumulate enough dissipated heat to raise the grinding surface temperature to the point that allowed PS to undergo its glass transition. Thus, no cohesive state was observed in prior studies on PS degradation by shaker milling, as those studies employed small steel spheres and jars and implemented convective cooling on the reactor exterior where larger spheres were used [21, 22].

### 3.2 Impact of Reaction Microenvironment on Depolymerization Rates

**FIGURE 3:**
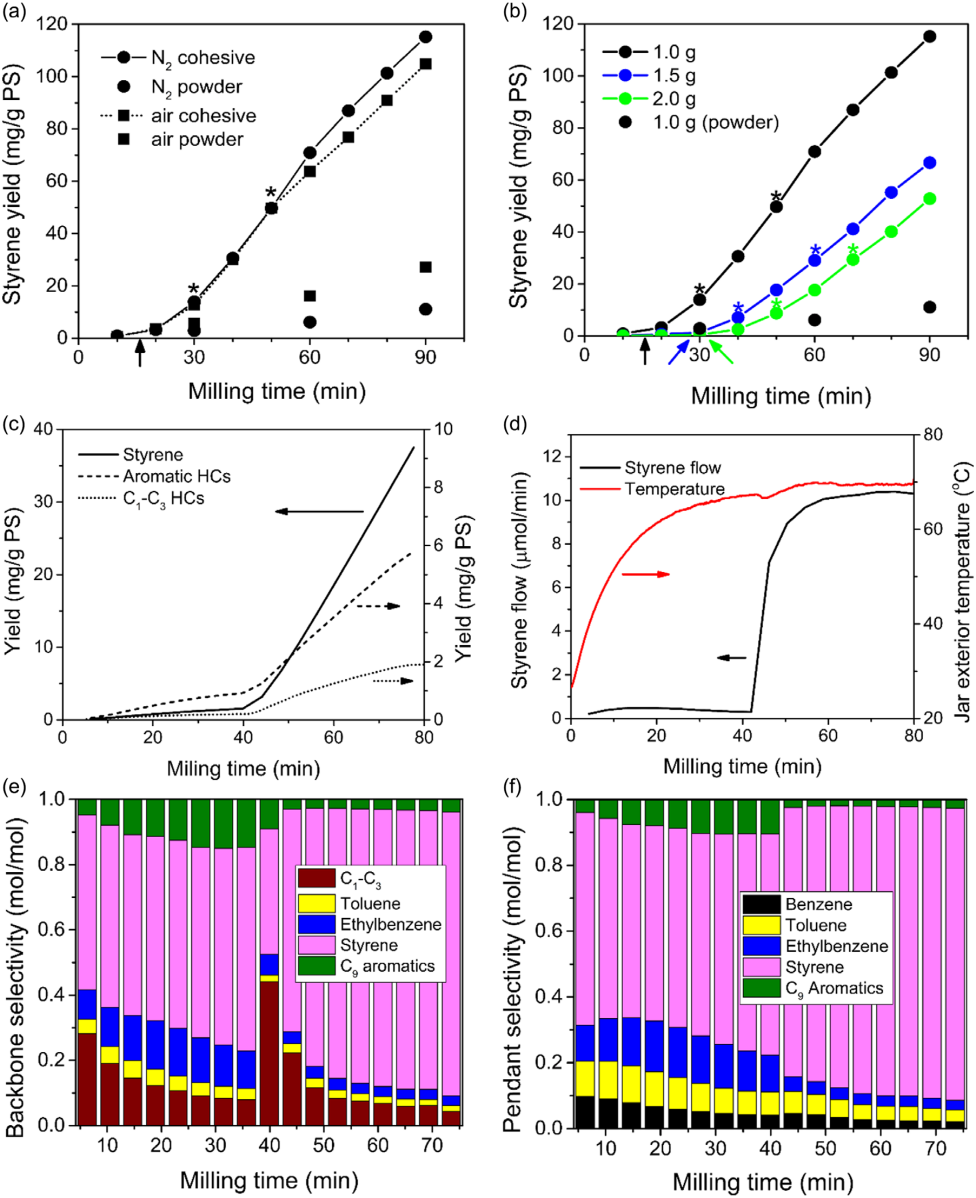
Cumulative yields for PS milled at 30 Hz employing eight 10 mm diameter WC spheres in a 25 mL WC reactor. Discrete sampling data include (a) 1 g of PS milled in N_2_ and air atmospheres with and without formation of the cohesive state (Table 1, Exp. 1 and 5–7) and (b) various amounts of PS milled in N_2_ (Table 1, Exp. 1–3) with formation of the cohesive state (powder milling for comparison). Color‐coded arrows indicate approximate time of cohesive state transition. Asterisks denote the sampling interval used to calculate initial rate in cohesive state as reported in Table 1. Continuous sampling data collected for conditions corresponding to Exp. 1 (Table 1) include (c) yields of styrene, aromatic byproducts and C_1_–C_3_ hydrocarbon gas byproducts, (d) styrene flow (note that in cohesive state milling the flow is unsteady for the first 10–15 min after transition) and reactor exterior temperature, and selectivities with time calculated with respect to (e) backbone carbon atoms (with methane and toluene counting for one unit of product; ethane, ethene, ethylbenzene and styrene count for two; benzene counts for zero, etc.) and (f) pendant phenyl groups (neglecting C_1_–C_3_ gases) on PS.

Discrete sampling experiments showed that styrene monomers produced by mechanochemical PS depolymerization inside the shaker mill reactor can be removed via a purge gas stream, collected downstream, and quantified to obtain cumulative yields with respect to milling time (Figure 3a,b). As in previous work with steel spheres, the presence of O_2_ enhanced the depolymerization of PS powder milled with WC (Figure 3a, dots versus squares) [22]. The initial rate of styrene production from PS powder in air was 2.6 times the rate obtained under N_2_ (Exp. 6 and 7, respectively, in Table 1).

Compared to baseline yields for PS powder, the formation of the cohesive state caused a dramatic increase in styrene production during milling in both air and N_2_ (Figure 3a). MW distributions (MWDs) of PS residues (Figure S4a) also showed that residues recovered in the cohesive state were in a more advanced state of degradation compared to powder residues that had undergone the same duration of milling. Upon formation of the cohesive state, styrene yield reached 60 mg/g PS in under 60 min at conditions of Exp. 1. The initial reaction rate established after transformation to the cohesive state was calculated using three data points (delineated by asterisks) spanning a 20‐minute interval that represents the steady‐state reactivity of PS in the cohesive state. Continuous sampling (see Ball Milling Experiments, Experimental Section) was implemented using an alternative setup under the same conditions as Exp. 1 (Table 1), which confirmed that a stable initial rate in the cohesive state can persist up to 30 min after formation (Figure S2a depicting instantaneous styrene flow), and that the physical transition from powder to a cohesive state occurred almost instantaneously with a significant increase in the yields of volatile products (Figure 3c) during two consecutive data points separated by only 4 min. On the other hand, the appearance of the cohesive state occurred at over 40 min into milling on the continuous sampling reactor setup versus 16 min on the discrete sampling setup, which can be attributed to differences in the environmental conditions around the reactor exterior between setups causing temperature development inside the reactor to differ. Specifically, the continuous sampling reactor was housed in a much larger enclosure that allowed for greater heat transfer away from the reactor exterior by passive convection.

By comparing Exp. 1 and 6 in Table 1, it is apparent that the rate of styrene production in the cohesive state was one order of magnitude (~12 times relative to powder in N_2_) higher than that of PS powder. Real‐time measurement of the exterior jar temperature indicated a rise from ambient temperature up to an initial plateau point at 70°C, with the temperature plateau coinciding with the sudden jump of about one order of magnitude in styrene flow to a sustained higher rate according to the continuous sampling experiment (Figure S3d). This corroborates the initial rate of styrene production in the cohesive state calculated in discrete‐sampling experiments. The attainment of steady‐state exterior temperature confirms that a thermal steady state of the system was achieved through a balance between internal heat generation from dissipation of kinetic energy in collisions, heat conduction through the reactor wall, and exterior heat exchange with the ambient atmosphere.

**FIGURE 4:**
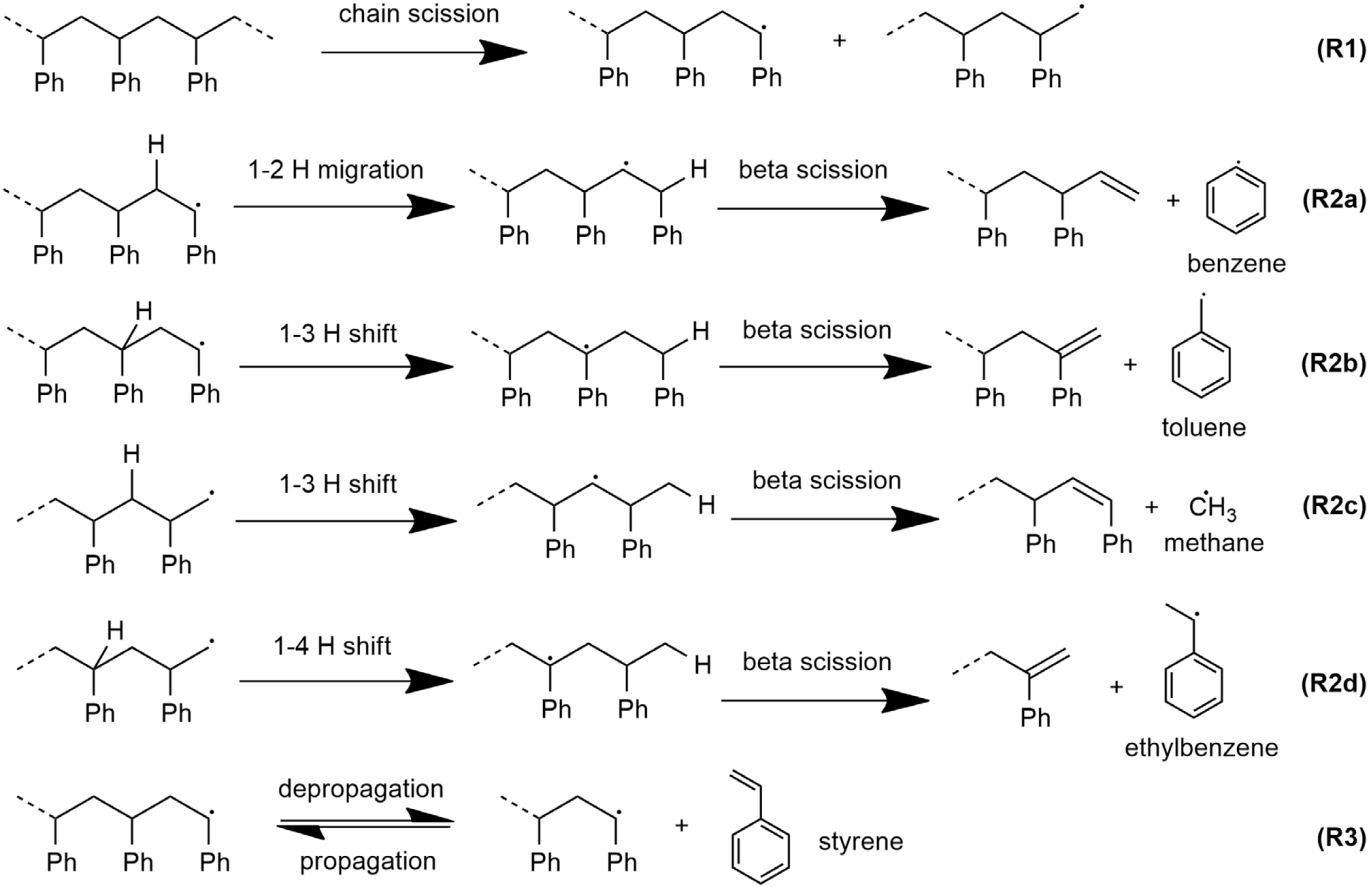
Elementary reactions relevant to the mechanistic discussion.

For the base case of PS milled in N_2_, the kinetic data obtained from continuous sampling (Figure S3c–f) showed the formation of the same byproducts in powder or cohesive state milling, including of benzene, toluene, ethylbenzene, allylbenzene, plus trace amounts of α‐methylstyrene, n‐propylbenzene, cumene, and five C_1–_C_3_ hydrocarbon gases—methane, ethene, propene, ethane, and propane (Figure S2), which can be formed according to the reaction scheme in Figure 4. Of these, the four most abundant byproducts from milling in the cohesive state were methane, toluene, ethylbenzene, and benzene (Figure S2). There was a transient burst of significant production of C_1_–C_3_ products, particularly methane, in the 10‐min interval immediately following cohesive state formation (Figure S2b), which occurred faster than the increase in styrene production. This resulted in the high methane selectivity at 40–50 min of milling (Figure 3e). The peak in methane production during or shortly after the formation of the cohesive state can be explained with a population of free radical intermediates formed during the induction period. Once the feedstock becomes cohesive, these intermediates are converted releasing methane, but they appear to not be formed as readily in the cohesive state.

During the initial phase of powder milling, chain‐end radical species can be generated by chain scissions (R1 in Figure 4) during particle breakage, and they accumulate steadily within the PS particles [21, 22]. Since the cohesive state has much lower surface area than the powder state, even radical species formerly located on the surfaces of PS particles are likely buried in the bulk interior of the cohesive film spread over the mass of grinding spheres. ESR spectra (Figure S5) of PS residue (both milled under N_2_) show that some of the radical species formed during the milling process are persisent enough to be detected in ex situ measurements. However, it is likely many radicals will have decayed before this analysis.

These radicals in the cohesive film remain reactive under mechanical action, being susceptible to elementary reactions found under pyrolysis conditions, such as depropagation (yielding styrene), beta scissions, and intramolecular hydrogen shifts [21, 34]. From an isolated secondary chain end radical, one hydrogen shift followed by one beta scission reaction is sufficient to form a radical intermediate that can form either benzene or toluene (Figure 4, R2a‐b). Likewise, one hydrogen shift plus one beta scission from the primary chain end radical are sufficient to produce methane and ethylbenzene (Figure 4, R2c‐d). It is known that most hydrogen shift steps have lower activation barriers compared to mid‐chain scissions beta scission [35]. Thus, hydrogen transfer reaction in the polymeric radicals will be more prevalent than C–C scission reaction in the low‐energy environment in the powder state. After one or more hydrogen transfer steps, the small molecules byproducts are generated in their radical form, but due to greater mobility compared to PS chain segments, they can easily abstract a hydrogen atom from the surrounding chain segments around the reactive radical site. The pathways leading to the byproducts leave behind an olefinic chain end that cannot produce another one of these same small molecules. Once the cohesive state is reached, the more energetic environment facilitates the more demanding beta‐scission steps, which increase the rates of formation of styrene and byproducts, according to the R4 and R2a‐d in Figure 4, respectively. However, under these conditions, newly formed radicals are more likely to undergo beta‐scission relative to hydrogen shift, which explains why the rate of formation of the byproducts declined within 10 min of the cohesive state's formation (Figure S2), whereas styrene flow maintained a steady level well after its establishment (Figure 3d).

The insensitivity of rate of styrene in the cohesive state production to O_2_, which was nearly identical for milling under N_2_ and air (Table 1, Exp. 1 and 5, respectively), is consistent with the notion that radicals located in the bulk interior of the cohesive film are mainly responsible for depolymerization. Consistent with this, the MWDs (Figure S4a) of cohesive residues milled under air and N_2_ exhibited greater similarity with each other compared to the distributions of the powder residues. In the latter case, powder milled in air exhibited noticeably greater degradation than in N_2_ as O_2_ was able to react with radicals on the much larger surface of the powder. For PS powder, O_2_ enhances monomer production by stabilizing specific chain end radical intermediates that favor depolymerization. Since O_2_ facilitates depolymerization through a surface‐chemical mechanism aided by the creation of fresh PS surfaces populated by chain‐end radicals through particle breakage [21, 22], this mechanism is more applicable to high‐surface area PS powder than the low‐surface area cohesive bulk film, so the absence of any apparent oxygen effect on cohesive state kinetics is consistent with the inapplicability of particle breakage to the dynamical behavior of the cohesive state.

As previously mentioned, the grinding surface temperature during powder state milling was only about 50°C under the specific conditions used here [21]. Meanwhile, temperatures were around 100°C during cohesive state milling. Thus, it is worthwhile to consider whether the reactor temperature difference of 50°C between powder and cohesive states can account for the increase in the styrene flow by a factor of 12 for the base case with N_2_ flow. Assuming the depropagation step (R3 in Figure 4) yielding monomer from a secondary chain end radical is thermodynamically limited, we may use a model [36] (section S.F., Supporting Information) of mechanochemical PS depolymerization at these conditions to calculate the gas phase monomer activity (which is proportional to monomer concentration) as a function of local temperature in the depolymerization zone of impacted PS. At 50°C, the styrene activity is 0.0036. For this activity to be amplified by a factor of 12 to the value 0.0432, a local temperature of 113°C would be needed. At exactly 100°C, the monomer activity is 0.0279, which is 7.7 times the activity at 50°C. Since the temperature measurements of 100°C were taken 20–30 s after the instant when milling was stopped (to dismount the jar from the mill), and during that same interval of time the accumulated heat from collisions was dissipating continuously away from the grinding surfaces, it is possible that while the mill was in operation, the local temperature of the grinding surfaces in contact with PS may have been as high as 113°C. Therefore, the calculated activity values for a temperature difference of 50–60°C in the reactor microenvironment are consistent with the observed quantity of monomer extracted from the ball mill per unit time differing by about an order of magnitude in the powder and cohesive states. However, this analysis only compares two homogeneous steady states and disregards that the temperature increase inside the reactor occurred before the significant increase in styrene flow (Figure 3d). Thus, it is suggested that heterogeneities between local reaction environments appear to significantly affect the kinetics as discussed below.

When increasing initial amounts of PS (1.0–3.0 g) were milled under the same set of conditions (Table 1, Exp. 1–4), the cohesive state formed later, and no cohesive state was formed with 3.0 g of PS up to 120 min of milling. At a higher filling degree of polymer particles, restricted ball motion reduces the kinetic energy of grinding spheres during milling, while a cushioning effect by these numerous particles distributes the impact energy over a larger amount of the feedstock. This leads to less energetic reaction environments even though the frequency of collisions is kept approximately constant when using the same milling frequency and configuration of grinding spheres [37, 38]. At the same time, a larger quantity of PS must be heated to the conditions necessary for the cohesive state to emerge. This explains why an increase in PS loading from 1.0 to 2.0 g led to progressively longer incubation times to accumulate the heat necessary to induce the cohesive state transition in PS (Figure 3b). Simultaneously, part of the energy dissipated through collisions with the reactor wall is thermally conducted through the wall into the exterior surroundings until a steady state is established (temperature curve in Figure S2b). Thus, beyond a certain PS loading, the energy dissipation from collisions should be sufficiently damped by particle cushioning, such that a given configuration of spheres is unable to attain a steady state grinding surface temperature high enough to induce the cohesive state transition. With eight 10 mm spheres, this occurred at a PS loading of 3.0 g. Consequently, PS remained in the powder state in perpetuity.

After cohesive state formation, initial rates calculated using the first three data points following cohesive state formation (Table 1, Exp. 1–3) decreased with increasing loading of PS from 1.0 to 2.0 g. MWDs of PS residues in the cohesive state indicated slightly less advanced degradation with increasing reactor loading consistent with the trend in the initial rates. Since higher loading means a greater overall mass of PS is distributed over the surface of the same mass of grinding spheres in the cohesive state, it can be inferred that the spheres are congealed together by a thicker layer of continuous PS, as is visually apparent from a comparison of Figure 1c and d. This implies that when loadings are higher, the kinetic energy transferred during the collisions of a constant mass of spheres is absorbed by a larger mass of PS, and each unit mass of PS receives a smaller dose of the mechanical energy available to drive depolymerization reactions. The result is reduced mass‐normalized initial styrene formation rates in the cohesive state with increasing loadings of PS. However, the absolute initial rates in the cohesive state were 1.67, 1.65, and 2.02 mg/min for PS loadings of 1.0, 1.5, and 2.0 g, respectively, which do show the approximate trend (within the error margin of ±0.1–0.2) that more reactions occur when the amount of cohesive reactant is increased. Since the spheres are congealed together by cohesive PS and travel as a single mass during milling regardless of loading, the absolute rates for the cohesive state calculated for experiments conducted at the same milling frequency should be comparable with each other as metrics for reactivity per collision.

There is a much sharper contrast in results between the experiments using different numbers of spheres with the same combined mass (Table 1, Exp. 8–10). Using one 19 mm sphere, two 15 mm spheres, or four 12 mm spheres, the total mass of spheres was 56.0, 55.1, and 56.5 g, respectively, which is close enough to render the kinetic energy during free flight between these configurations comparable. Using one 19 mm sphere, the cohesive state was formed 10 min earlier than with eight 10 mm spheres, and yet, styrene production remained much lower in the cohesive state when only one sphere was used. The initial rate in the cohesive state increased to 0.84 mg/g PS/min using two 15 mm spheres, and further to 2.65 mg/g PS/min using four 12 mm spheres—the highest recorded rate of all configurations investigated. These drastically different initial rates were seen despite the formation of the cohesive state, indicating that the same elevated temperature (~100°C) was attained inside the jar with any of these configurations of spheres. With four 12 mm spheres, the initial rate in the cohesive state was also just under 12 times the initial rate in the powder state, in accordance with the magnitude of difference in monomer activity at two temperatures 50°C apart predicted by thermodynamic considerations discussed several paragraphs ago.

**FIGURE 5:**
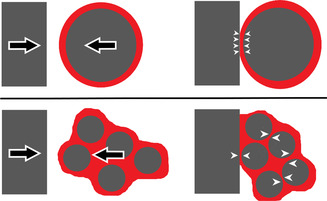
Two‐dimensional schematics contrasting a collision involving one single sphere (gray) coated in cohesive state PS (red) versus a collection of several spheres congealed by cohesive PS. A collision involving the latter configuration contains internal shearing dynamics not present in the former. White arrowheads represent illustrative stress vectors acting on patches of the cohesive film sandwiched between grinding surfaces at closest separation during a collision.

These results offer insight into the type of grinding forces that are not conducive to driving depolymerization reactions. The single 19 mm sphere was coated by a macroscopically homogeneous shell of roughly uniform thickness (Figure 1b), which is expected to be a soft, viscous layer of PS during mill operation at the polymer's glass transition range. The kinematic behavior of single spheres agitated in a shaker mill jar is well‐characterized [39, 40], and mostly consists of compressive, sometimes head‐on collisions between the sphere and the jar wall. This kinematic behavior should apply to the same sphere engulfed within a layer of viscous polymer. Thus, it is reasonable to assume that the cohesive state formed around one 19 mm sphere would be primarily subjected to compressive forces in the contact region between sphere surface and wall during collisions, as depicted in the top schematic of Figure 5. Measurements of styrene production indicate that this configuration of collisions is relatively ineffective at driving depolymerization reactions in the cohesive PS state (Table 1, Exp. 8).

By contrast, with two or more spheres, the colliding body is a loose collection of solid spheres held together by a viscous continuous link or network of PS. In free flight, the congealed collection of spheres may be regarded as a single rigid body, and its average kinematic behavior may mimic that of a single sphere during milling. However, the dynamics of a loosely congealed body during the course of a collision with the jar wall are expected to be markedly different. Rather than a single well‐defined contact region that experiences mostly unidirectional compression during the contact time of collision, the loosely congealed body likely deforms in a fluid‐like manner with relative internal motion (such as frictional shearing) between macroscopic constituent parts of the body, leading to significant changes in its shape (Figure 5, bottom). In such a configuration, it is reasonable to conjecture that shear forces play a more significant role in portions of the colliding body. The significance of shear forces is expected to increase going from one 19 mm sphere to 2, 4, or 8 smaller spheres. This matches the same qualitative trend of increasing initial rates of the cohesive states depicted in Table 1, Exp. 8–10 with increasing number of spheres (holding total sphere mass constant). The correlation between these two trends allows us to convincingly suggest that the kinetics of the elementary depropagation reaction causing styrene production is primarily determined by the shear forces experienced by cohesive PS during collisions. In contrast, compressive forces acting on the cohesive layer contribute negligibly to the production of styrene. As a result, a cohesive layer of PS that experiences primarily compressive forces produces styrene at a limited rate, despite being at an elevated temperature on the glass transition range of PS that enhances the thermodynamic favorability of depolymerization. Instead, macroscopic shearing motion apparently correlates to highly reactive environments surrounding mechanoradicals at the molecular level, allowing for enhanced styrene production through depropagation from these radicals.

## 4 Conclusion

This work explores the kinetics of mechanochemical PS depolymerization when ball‐milled in the form of a cohesive state. This cohesive film coats the grinding balls and forms spontaneously from PS powder when the temperature of the grinding surfaces in the mill approaches the glass transition range of PS. Air cooling the mill exterior during operation can suppress the formation of the cohesive state by reducing the steady‐state temperature of grinding surfaces inside the reactor. This demonstrates that small design alterations of the milling equipment that allow for passive temperature development to characteristic temperatures where a material undergoes state transitioning can be leveraged to induce the formation of highly active cohesive states.

In this cohesive state, the rate of monomer production increases by one order of magnitude compared to the kinetics exhibited by PS powder (Figure S2b). The temperature difference between the warmer cohesive state and colder powder state milling of PS reduces the thermodynamic limitation of styrene formation from a chain end radical species, but the relative timing of the increases of temperature and rate as well as the influence of the configurations of grinding spheres indicates the specific local reaction environments play a key role. Specifically, shear forces drive this reaction much more effectively than purely compressive ones. Variation of the reactant loading shows that styrene production critically depends on the density of mechanical energy input per unit mass of polymer.

The demonstration of enhanced kinetics via a cohesive state is especially useful for potential mechanochemical approaches towards depolymerizing other olefinic polymers that are challenging to chemically recycle/upcycle. In the case of PS, it is noteworthy that the conditions producing its reactive cohesive state are the same as those that lead to pronounced viscoelastic behavior [41, 42].

## Supporting Information

Additional supporting information can be found online in the Supporting Information section. Photographs of cohesive and powder residues of PS; additional data from continuous sampling experiment; MWDs and tabulated average MWs of select PS samples; ESR spectra of residues; time‐resolved intensity and frequency spectrograms of audio collected in the vicinity of the cohesive transition; exposition of thermodynamic model for depolymerization. **Supporting Fig. S1a:** WC, 1 g PS, 1 × 19 mm, N_2_ flow (left) coated sphere and (right) coating broken off. **Supporting Fig. S1b:** Steel, 1 g PS, 1 × 20 mm, N_2_ flow. The shiny steel sphere is partially encased in dark grey shell of PS residue sitting in the bottom half of the two‐piece jar. **Supporting Fig. S1c:** WC, 1 g PS, 2 × 15 mm, N_2_ flow. **Supporting Fig. S1d:** WC, 1 g PS, 4 × 12 mm, N_2_ flow. **Supporting Fig. S1e:** WC, 1 g PS, 8 × 10 mm, N_2_ flow. **Supporting Fig. S1f:** WC, 1.5 g PS, 8 × 10 mm, N_2_ flow. **Supporting Fig. S1g:** WC, 2 g PS, 8 × 10 mm, N_2_ flow. **Supporting Fig. S1h:** WC, 3 g PS, 8 × 10 mm, N_2_ flow. **Supporting Fig. S1i:** WC, 1 g PS, 8 × 10 mm, N_2_ flow, external cooling. **Supporting Fig. S1j:** 1 g PS, 10 × 10 mm, N_2_ flow, external heating (a) WC and (b) steel grinding tools. Spheres are relatively clean whereas patches of PS residue can be seen coated on the internal surface of the jar. **Supporting Fig. S2:** 1 g PS milled at 30 Hz in a WC reactor with eight 10 mm diameter WC spheres; instantaneous yields of (a) aromatic products (b) light hydrocarbon gases, equivalent to product flow multiplied by sampling interval (2.1 min for C_1_‐C_3_ gases and 4.2 min for aromatics). The left‐pointing arrow indicates the data series (styrene or methane) plotted according to the left vertical axis. All other data series are plotted according to the right vertical axis. **Supporting Fig. S3:** Reaction scheme of mechanochemical PS depolymerization in the cohesive state, in N_2_ atmosphere. **Supporting Fig. S4:** MWDs of residues recovered at 90 min from the same experiments comparing (a) N_2_ versus air, cohesive versus powder residues and (b) different PS loadings. **Supporting Fig. S5:** ESR spectra of powder and cohesive state residues milled in N_2_ for the same duration. **Supporting Fig. S6a:** Intensity spectrum and frequency spectrogram for an audio sampling of the ambient lab environment housing the ball mill reactor. **Supporting Fig. S6b:** Intensity spectrum and frequency spectrogram for an audio sampling of the ball mill equipped with empty grinding jars operating at 30 Hz. Compared to Figure S6a, the presence of a fundamental frequency band at 30 Hz and a weak overtone band at 60 Hz are noteworthy. **Supporting Fig. S6c:** Intensity spectrum and frequency spectrogram in vicinity of cohesive transition corresponding to first formation of a cohesive state under N_2_ with 8 × 10 mm spheres and 1.0 g PS (conditions detailed in Table 1 in main text as Exp. 1). See Figure S7 for an illustration of how this (and subsequent) audio clip was sampled from the raw audio recording data. **Supporting Fig. S6d:** Intensity spectrum and frequency spectrogram in vicinity of cohesive transition corresponding to second formation of a cohesive state (after pausing milling, cooling reactor down to ambient temperature and restarting milling) under N_2_ with 8 × 10 mm spheres and 1.0 g PS (conditions detailed in Table 1 in main text as Exp. 1). **Supporting Fig. S6e:** Intensity spectrum and frequency spectrogram in vicinity of cohesive transition corresponding to formation of a cohesive state under N_2_ with 8 × 10 mm spheres and 1.5 g PS (conditions detailed in Table 1 in main text as Exp. 2). **Supporting Fig. S6f:** Intensity spectrum and frequency spectrogram in vicinity of cohesive transition corresponding to formation of a cohesive state under N_2_ with 8 × 10 mm spheres and 2.0 g PS (conditions detailed in Table 1 in main text as Exp. 3). **Supporting Fig. S6g:** Intensity spectrum and frequency spectrogram in vicinity of cohesive transition corresponding to the first formation of a cohesive state under air with 8 × 10 mm spheres and 1.0 g PS (conditions detailed in Table 1 in main text as Exp. 5). **Supporting Fig. S6h:** Intensity spectrum and frequency spectrogram in vicinity of cohesive transition corresponding to second formation of a cohesive state (after pausing milling, cooling reactor down to ambient temperature and restarting milling) under air with 8 × 10 mm spheres and 1.0 g PS (conditions detailed in Table 1 in main text as Exp. 5). **Supporting Fig. S6i:** Intensity spectrum and frequency spectrogram in vicinity of cohesive transition corresponding to formation of a cohesive state under N_2_ with 1 × 19 mm sphere and 1.0 g PS (conditions detailed in Table 1 in main text as Exp. 8). **Supporting Fig. S6j:** Intensity spectrum and frequency spectrogram in vicinity of cohesive transition corresponding to formation of a cohesive state under N_2_ with 2 × 15 mm spheres and 1.0 g PS (conditions detailed in Table 1 in main text as Exp. 9). **Supporting Fig. S6k:** Intensity spectrum and frequency spectrogram in vicinity of cohesive transition corresponding to formation of a cohesive state under N_2_ with 4 × 12 mm spheres and 1.0 g PS (conditions detailed in Table 1 in main text as Exp. 10). **Supporting Fig. S7:** Screenshot of raw audio recording data corresponding to Figure S6c, illustrating how the processed data was clipped from the raw audio. Also indicated is visual identification of the cohesive transition as a sudden increase in intensity range at 16 min. (see Exp. 1, Table 1 in main text) into ball milling. **Supporting Fig. S8:** Schematic illustration of basic features of the steady‐state mechanochemical PS depolymerization system including relevant variables discussed in the model. **Supporting Fig. S9:** Depolymerization equilibrium constant for PS – equal to monomer activity *a*_M_ as a function of local temperature in depolymerization zone. **Supporting Table S1:** Number‐ (*M* *_n_*) and weight‐ (*M*_w_) average molecular weights of unmilled PS (with manufacturer‐reported *M*_w_ = 192,000 g/mol) and of residues from select experiments tabulated in Table S1 in the main text. All characterized residues were milled for 90 min at 30 Hz. Exp. # here corresponds to same identifier in the main text.

## Funding

This work was supported by the National Science Foundation (2028998); U.S. Department of Energy (KC0315010); Dutch Ministry of Education, Science and Culture; Dutch TKI Groene Chemie & Circulariteit; and Advanced Research Center (ARC) Chemical Buildings Blocks Consortium.

## Supporting information

Supplementary Material
