# Supplementary material for: Accelerated Mechanochemical Depolymerization of Poly(styrene) Due To Formation of a Cohesive State
Source: ChemSusChem. 2026 Apr 5;19(7):e70566. doi: 10.1002/cssc.70566 (PMC13050541; doi:10.1002/cssc.70566)
Supplement: Supplementary file 1 — Supplementary Material [file CSSC-19-e70566-s001.pdf]

Supporting Information for

ACCELERATED MECHANOCHEMICAL DEPOLYMERIZATION OF POLY(STYRENE) DUE TO FORMATION OF A COHESIVE STATE

Yuchen Chang,[a] Aubrey M. Hepstall,[a] Adrian H. Hergesell,[b] Claire L. Seitzinger,[b] Pawel Chmielniak,[a] Ina Vollmer,[b] and Carsten Sievers*[a]

[a] YC, AMH, PC, CS
School of Chemical and Biomolecular Engineering
Georgia Institute of Technology
311 Ferst Dr. NW, Atlanta, GA 30332, United States.
E-mail: carsten.sievers@chbe.gatech.edu

[b] AHH, CLS, IV
Inorganic Chemistry and Catalysis, Institute for Sustainable and Circular Chemistry
Utrecht University
Universiteitsweg 99, 3584 CG, Utrecht, The Netherlands.

Number of pages: 23
Number of figures: 9
Number of tables: 1

S.A. Photographs of Residues

Images were taken upon opening the jar after grinding at specified conditions. All grinding was conducted at 30 Hz in a 25 mL jar.

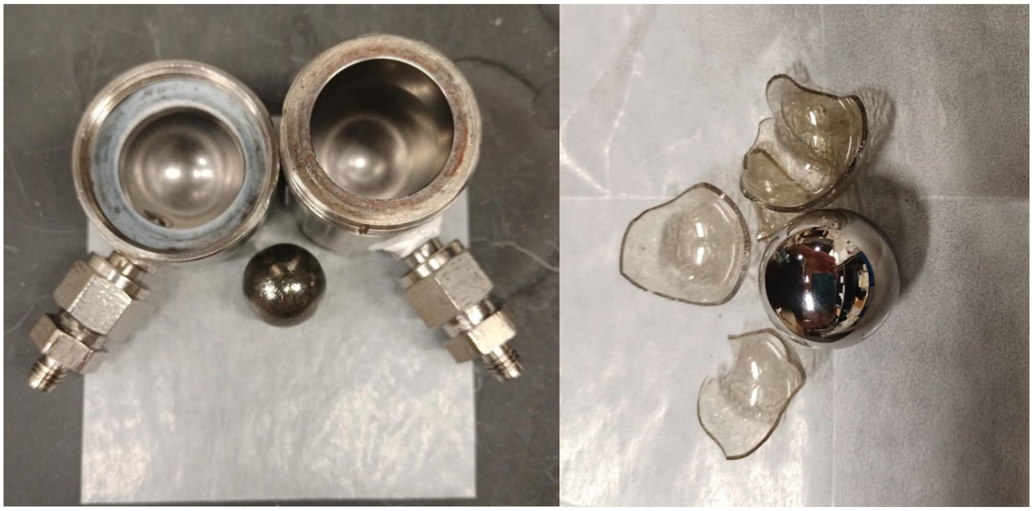

Figure S1a. WC, 1 g PS, 1x19 mm, N_2 flow (left) coated sphere and (right) coating broken off.

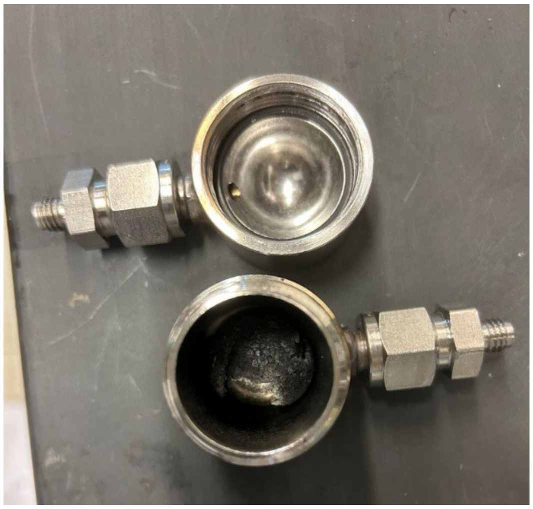

Figure S1b. Steel, 1 g PS, 1x20 mm, N_2 flow. The shiny steel sphere is partially encased in dark grey shell of PS residue sitting in the bottom half of the two-piece jar.

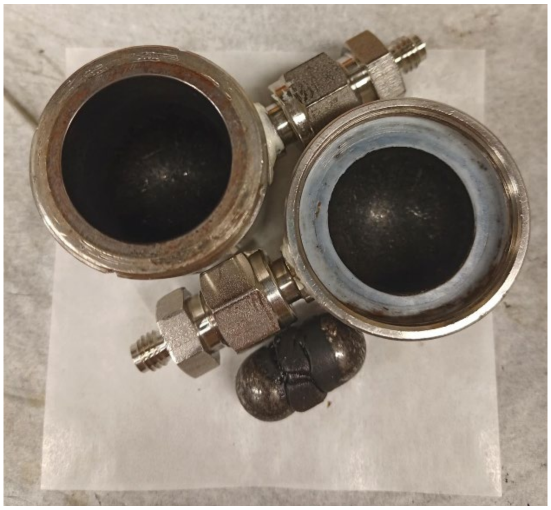

Figure S1c. WC, 1 g PS, 2x15 mm, N_2 flow.

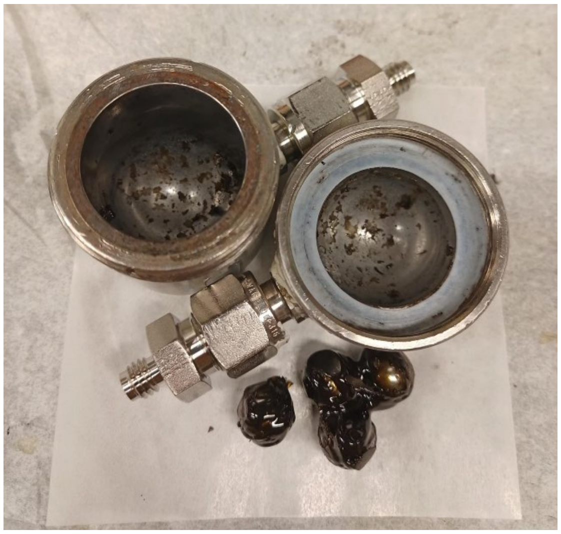

Figure S1d. WC, 1 g PS, 4x12 mm, N_2 flow.

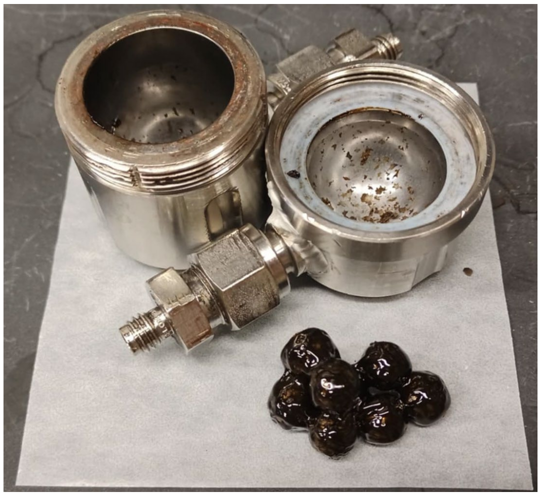

Figure S1e. WC, 1 g PS, 8x10 mm, N_2 flow.

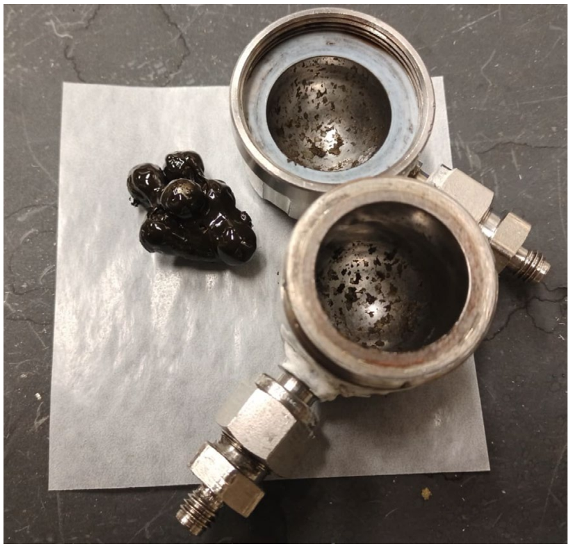

Figure S1f. WC, 1.5 g PS, 8x10 mm, N_2 flow.

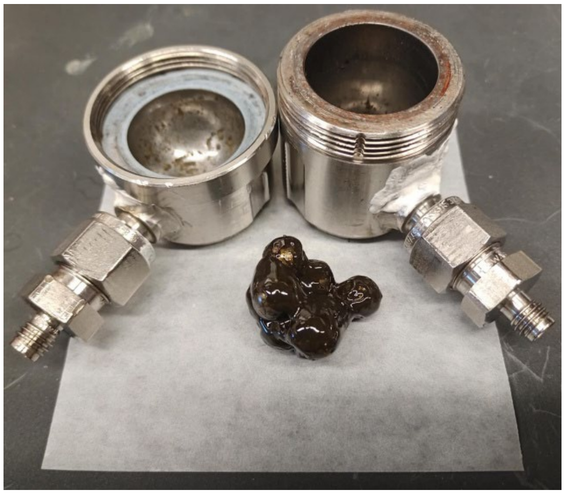

Figure S1g. WC, 2 g PS, 8x10 mm, N_2 flow.

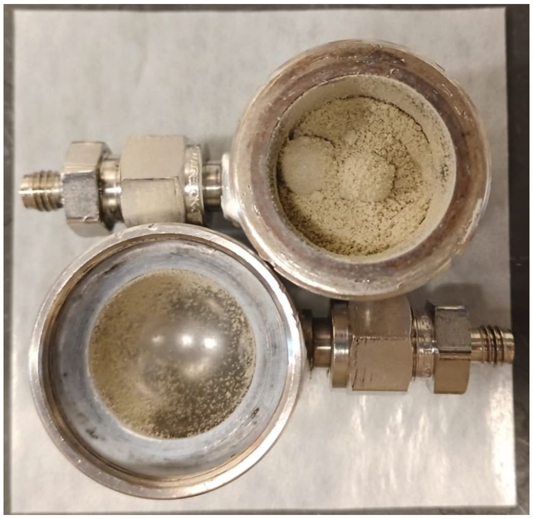

Figure S1h. WC, 3 g PS, 8x10 mm, N_2 flow.

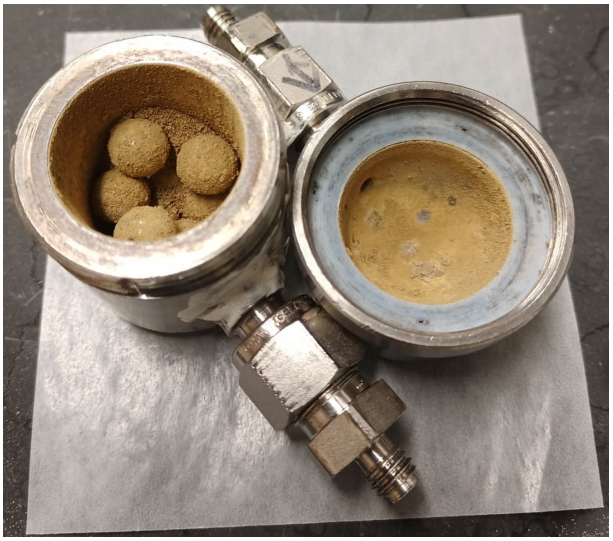

Figure S1i. WC, 1 g PS, 8x10 mm, N_2 flow, external cooling.

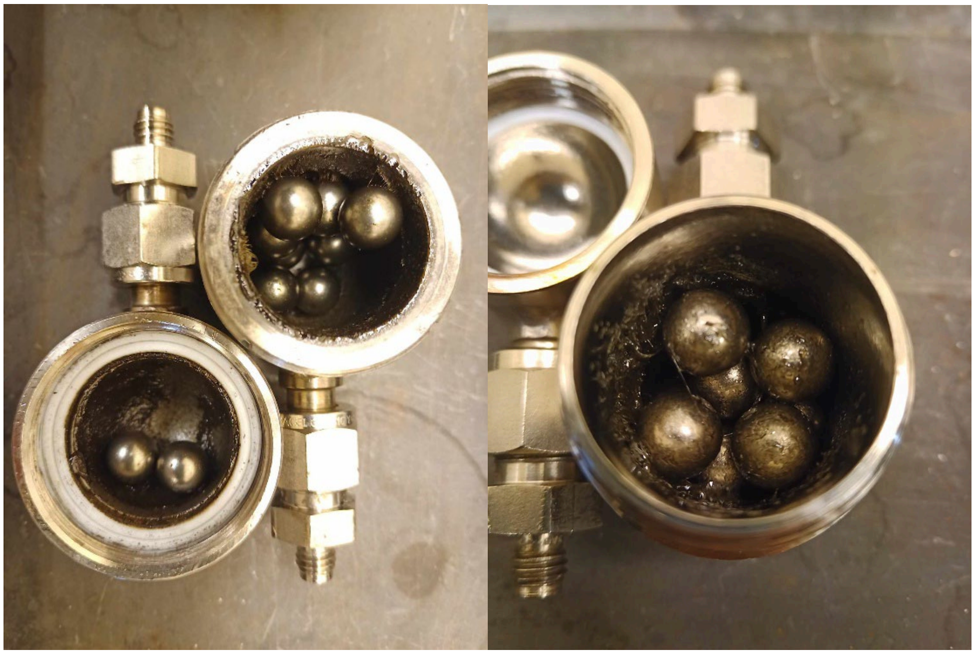

Figure S1j. 1 g PS, 10x10 mm, N2 flow, external heating (a) WC and (b) steel grinding tools. Spheres are relatively clean whereas patches of PS residue can be seen coated on the internal surface of the jar.

S.B. Continuous Sampling Experimental Data

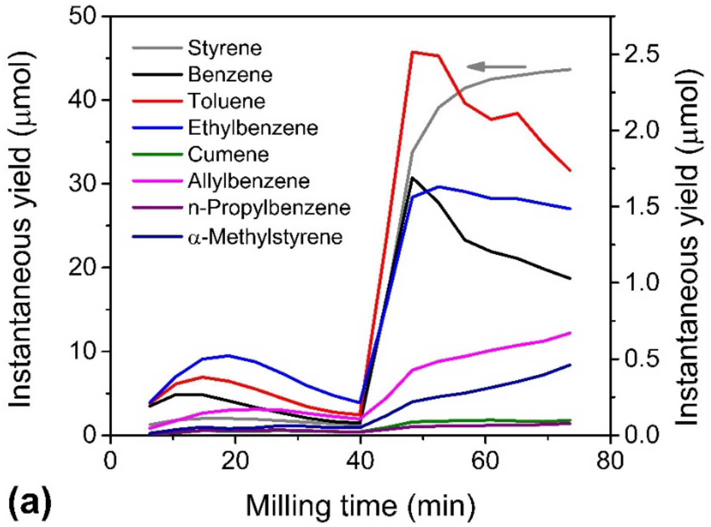

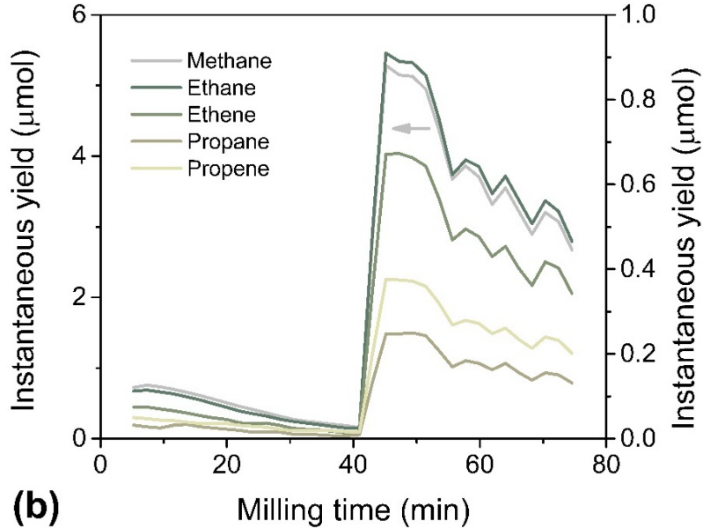

Figure S2. 1 g PS milled at 30 Hz in a WC reactor with eight 10 mm diameter WC spheres; instantaneous yields of (a) aromatic products (b) light hydrocarbon gases, equivalent to product flow multiplied by sampling interval (2.1 min for C_1-C_3 gases and 4.2 min for aromatics). The left-pointing arrow indicates the data series (styrene or methane) plotted according to the left vertical axis. All other data series are plotted according to the right vertical axis.

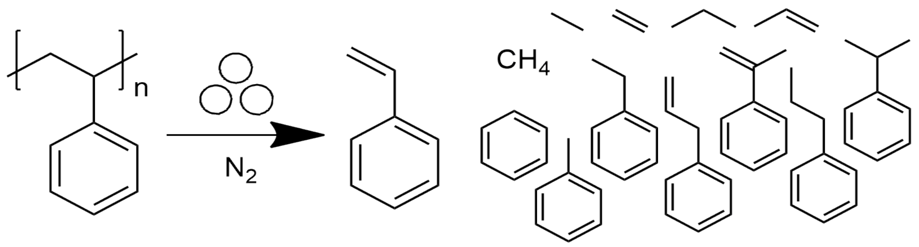

Figure S3. Reaction scheme of mechanochemical PS depolymerization in the cohesive state, in N_2 atmosphere.

S.C. Size Exclusion Chromatography (SEC)

Molecular weight distributions (MWDs) of solid residues recovered at the end of milling were obtained using size-exclusion chromatography (SEC) performed on a Tosoh EcoSEC HLC-8320GPC equipped with TSKgel SuperMultipore HZ-M column, internal refractive index detector (RID) and Wyatt Technology DAWN8+ dynamic light scattering detector (DLS), operating at 40°C. The eluent was chloroform containing 0.3% triethylamine at a flow rate of 0.45 mL/min and samples were prepared at concentrations of 5 to 10 mg/mL in the eluent.

Table S1. Number- (M_n) and weight- (M_w) average molecular weights of unmilled PS (with manufacturer-reported M_w = 192,000 g/mol) and of residues from select experiments tabulated in Table 1 in the main text. All characterized residues were milled for 90 min at 30 Hz. Exp. # here corresponds to same identifier in the main text.

Exp. #	Initial Mass (g) of PS (in exp.)	Gas phase	Cooling	M_n	M_w	Dispersion
Unmilled	n.a.	n.a.	n.a.	85,873	213,867	2.490
1	1.0	N_2	No	9,492	19,353	2.039
2	1.5	N_2	No	13,206	26,848	2.033
3	2.0	N_2	No	15,978	33,732	2.111
5	1.0	Air	No	8,872	16,569	1.868
6	1.0	N_2	Yes	13,336	22,858	1.714
7	1.0	Air	Yes	11,188	16,866	1.276

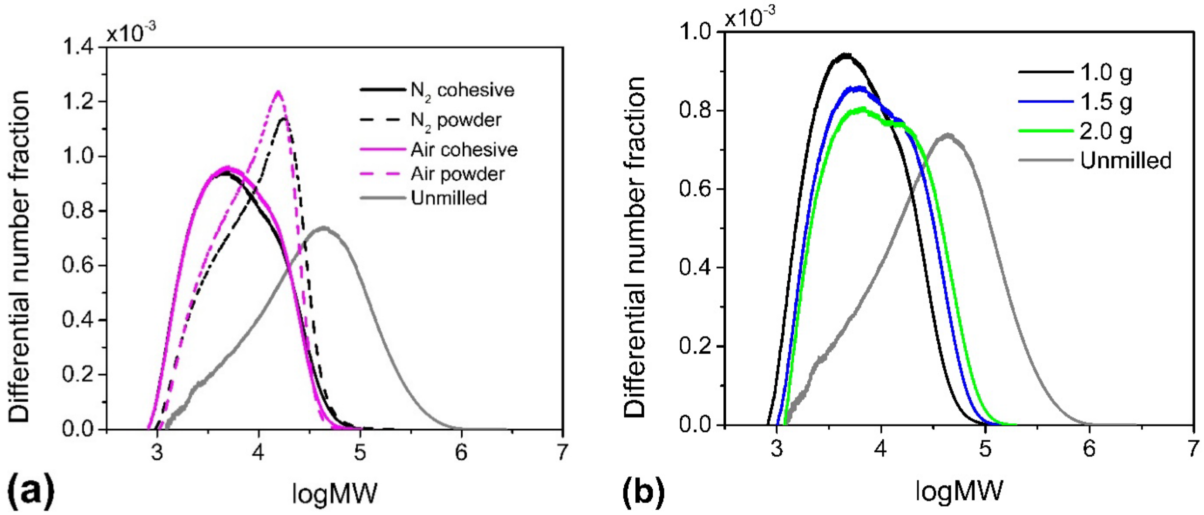

Figure S4. MWDs of residues recovered at 90 min from the same experiments comparing (a) N_2 versus air, cohesive versus powder residues and (b) different PS loadings.

S.D. Electron Spin Resonance (ESR) Spectra

Electron spin resonance (ESR) spectroscopy was conducted on residues using a Bruker EMXplus instrument at an X-band microwave frequency of 9.4 GHz typically using a modulation frequency of 1 G, at ambient temperature.

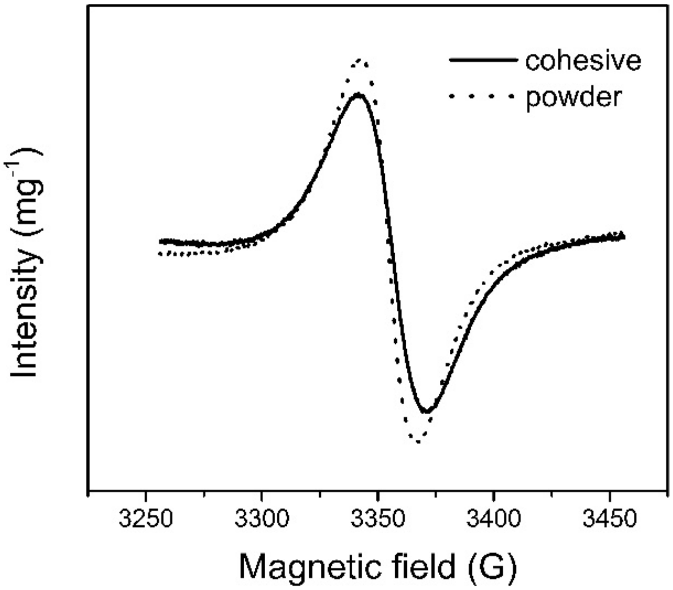

Figure S5. ESR spectra of powder and cohesive state residues milled in N_2 for the same duration.

S.E. Audio Spectral Analyses

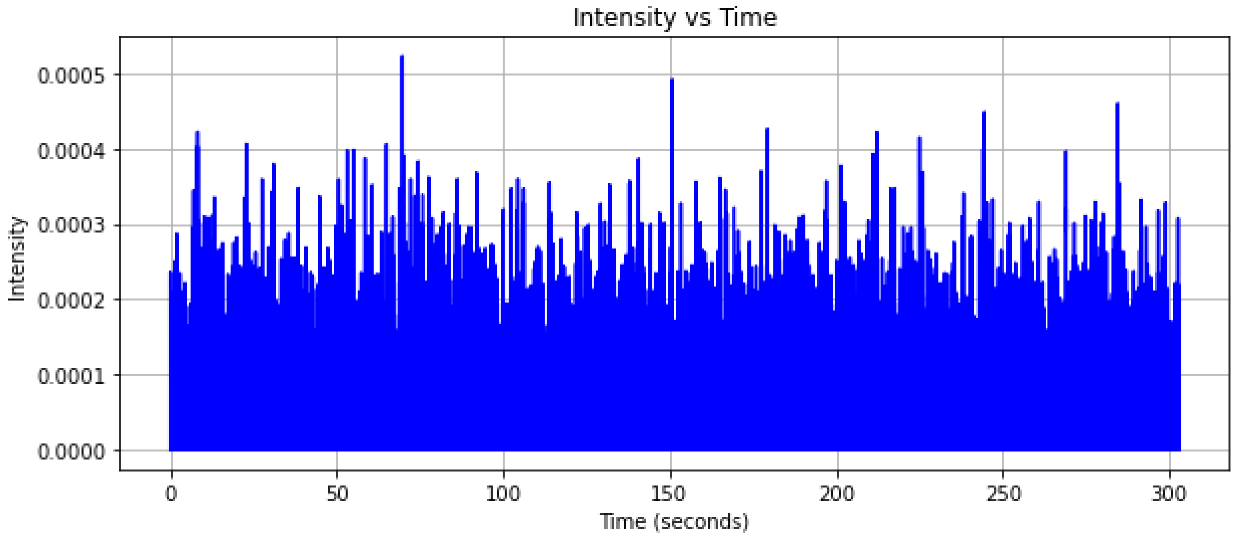

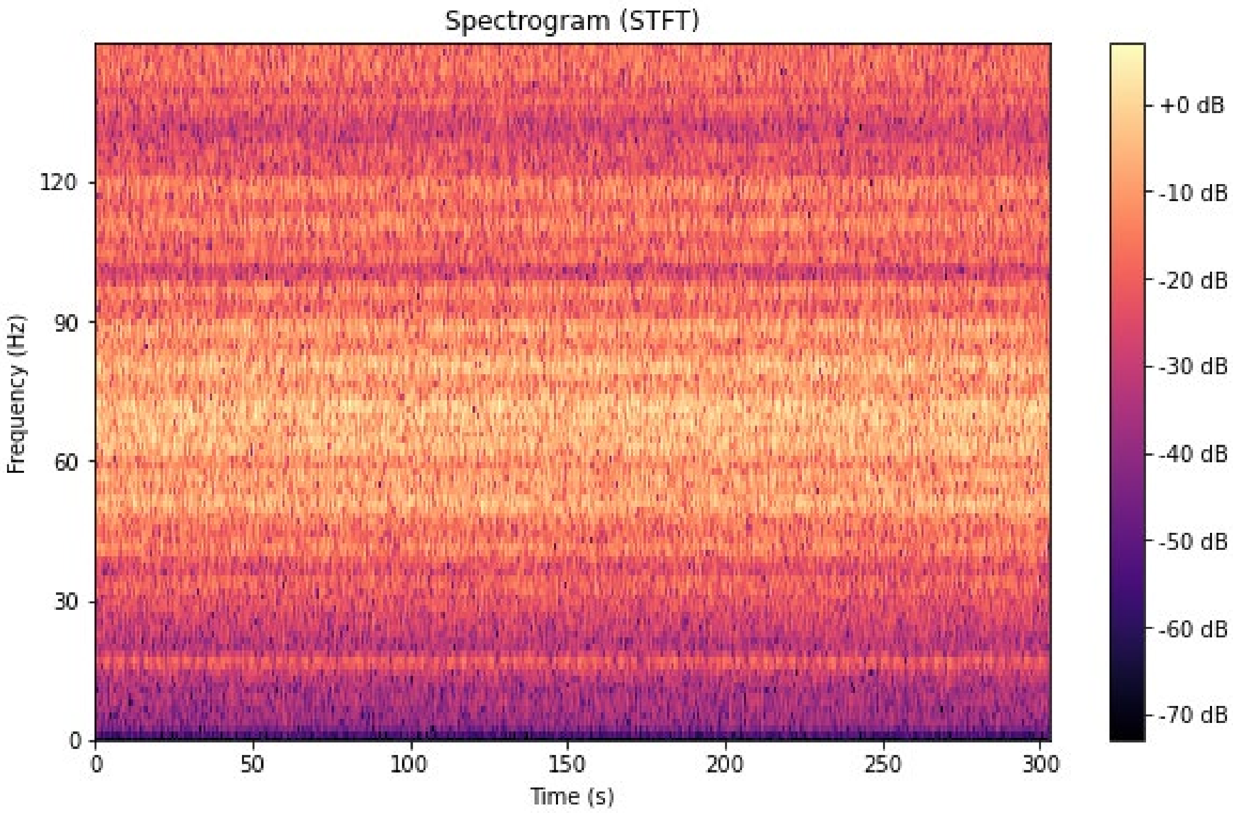

Figure S6a. Intensity spectrum and frequency spectrogram for an audio sampling of the ambient lab environment housing the ball mill reactor.

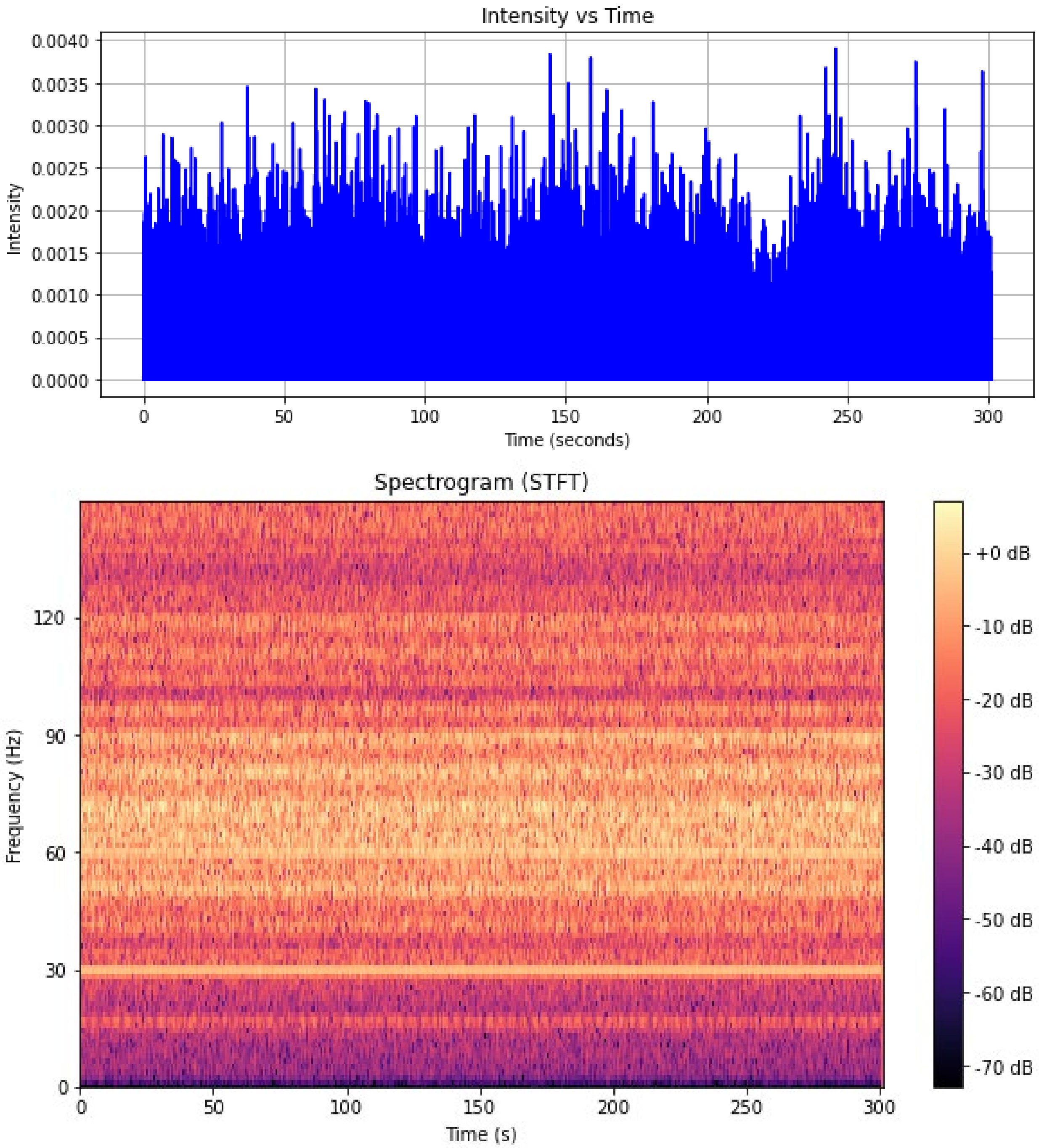

Figure S6b. Intensity spectrum and frequency spectrogram for an audio sampling of the ball mill equipped with empty grinding jars operating at 30 Hz. Compared to Figure S6a, the presence of a fundamental frequency band at 30 Hz and a weak overtone band at 60 Hz are noteworthy.

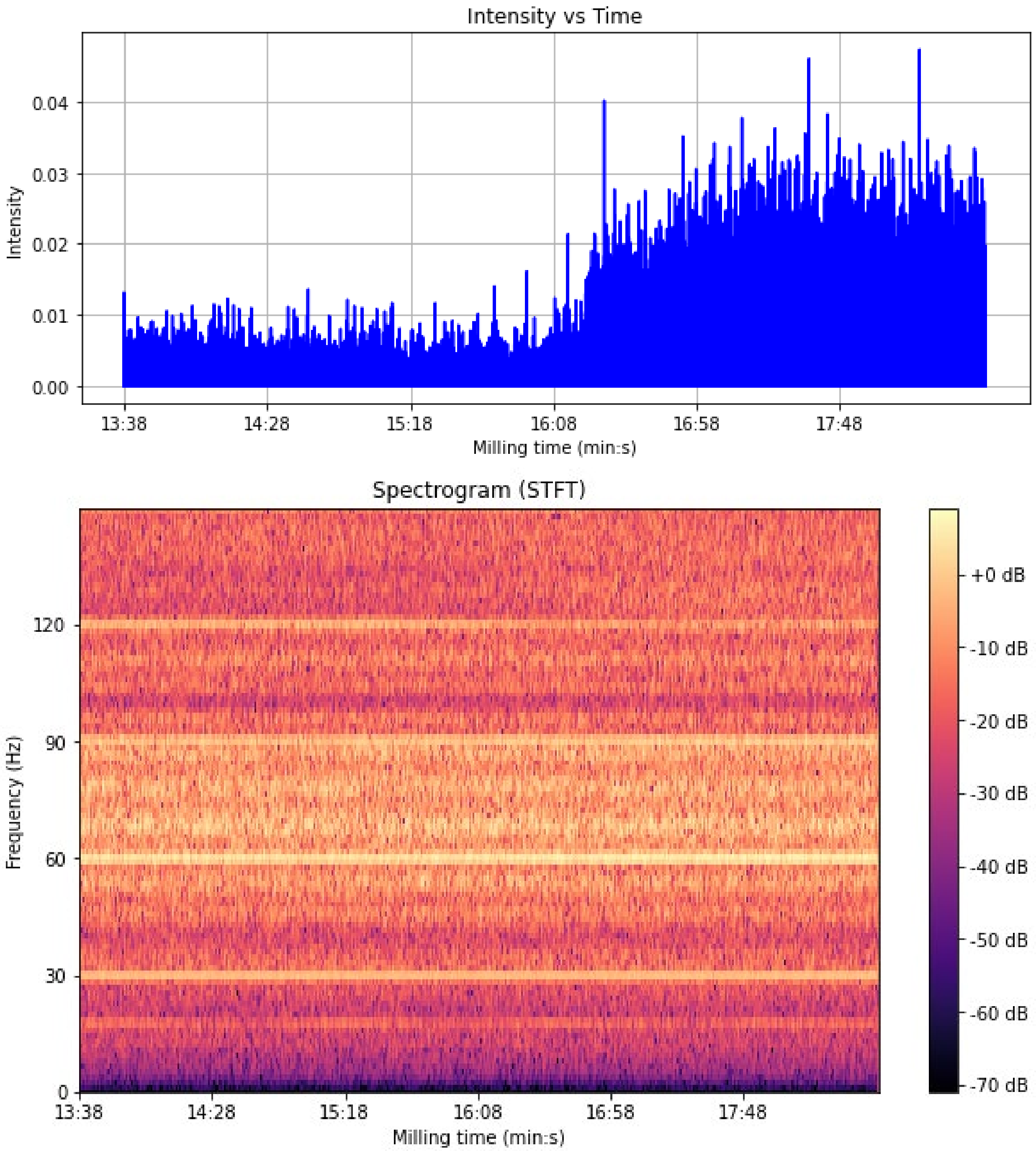

Figure S6c. Intensity spectrum and frequency spectrogram in vicinity of cohesive transition corresponding to first formation of a cohesive state under N_2 with 8 x 10 mm spheres and 1.0 g PS (conditions detailed in Table 1 in main text as Exp. 1). See Figure S7 for an illustration of how this (and subsequent) audio clip was sampled from the raw audio recording data.

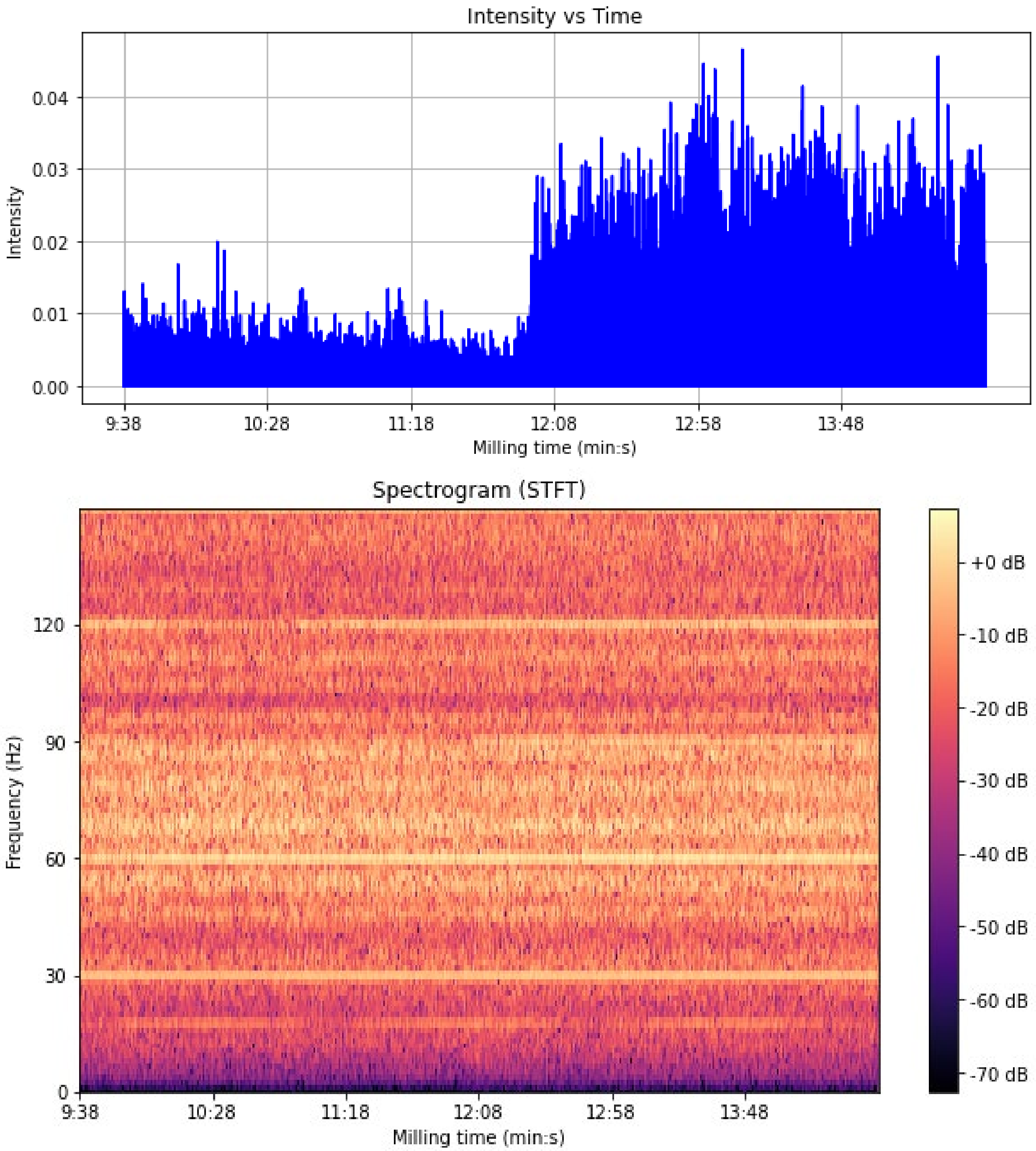

Figure S6d. Intensity spectrum and frequency spectrogram in vicinity of cohesive transition corresponding to second formation of a cohesive state (after pausing milling, cooling reactor down to ambient temperature and restarting milling) under N_2 with 8 x 10 mm spheres and 1.0 g PS (conditions detailed in Table 1 in main text as Exp. 1).

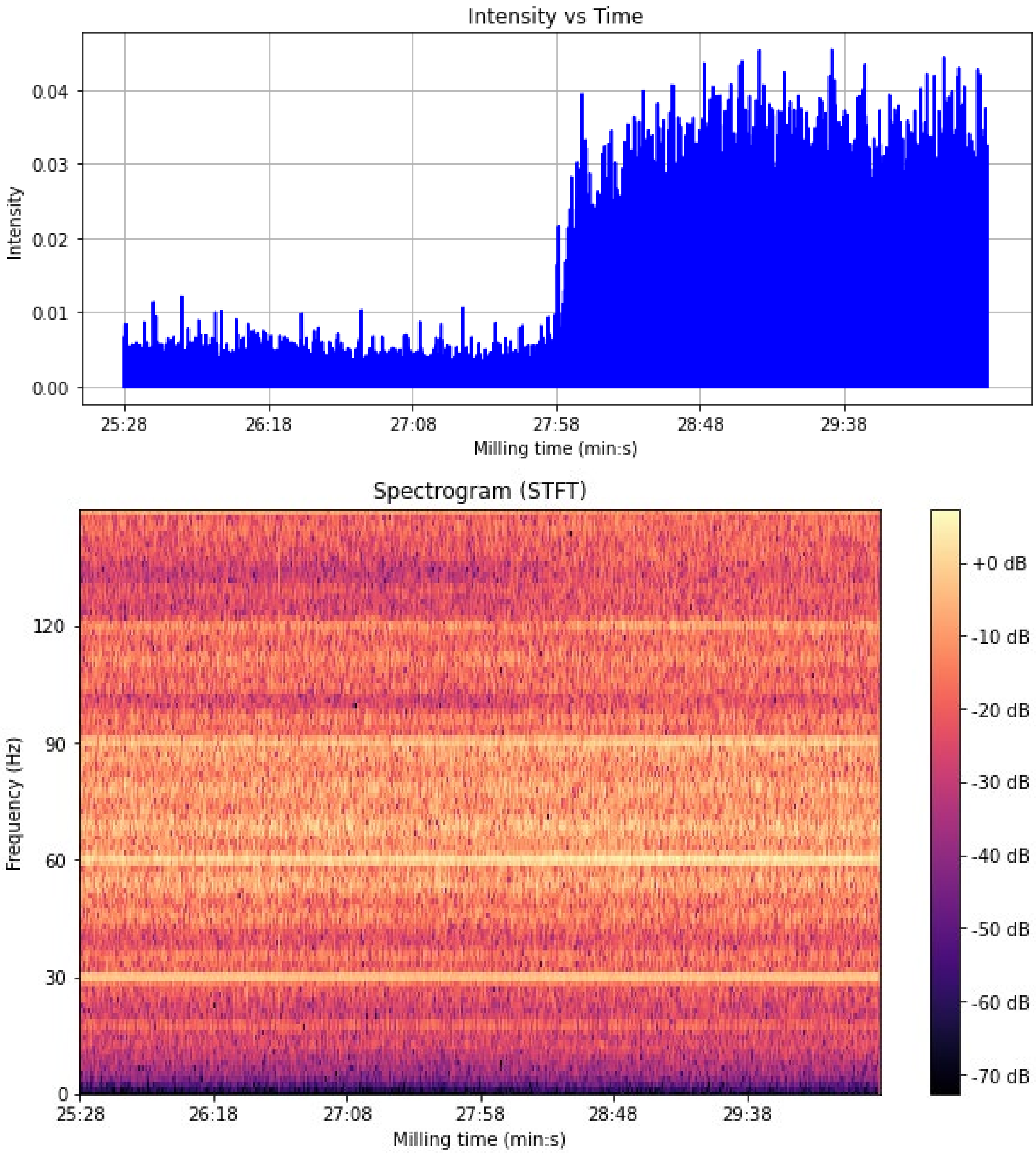

Figure S6e. Intensity spectrum and frequency spectrogram in vicinity of cohesive transition corresponding to formation of a cohesive state under N_2 with 8 x 10 mm spheres and 1.5 g PS (conditions detailed in Table 1 in main text as Exp. 2).

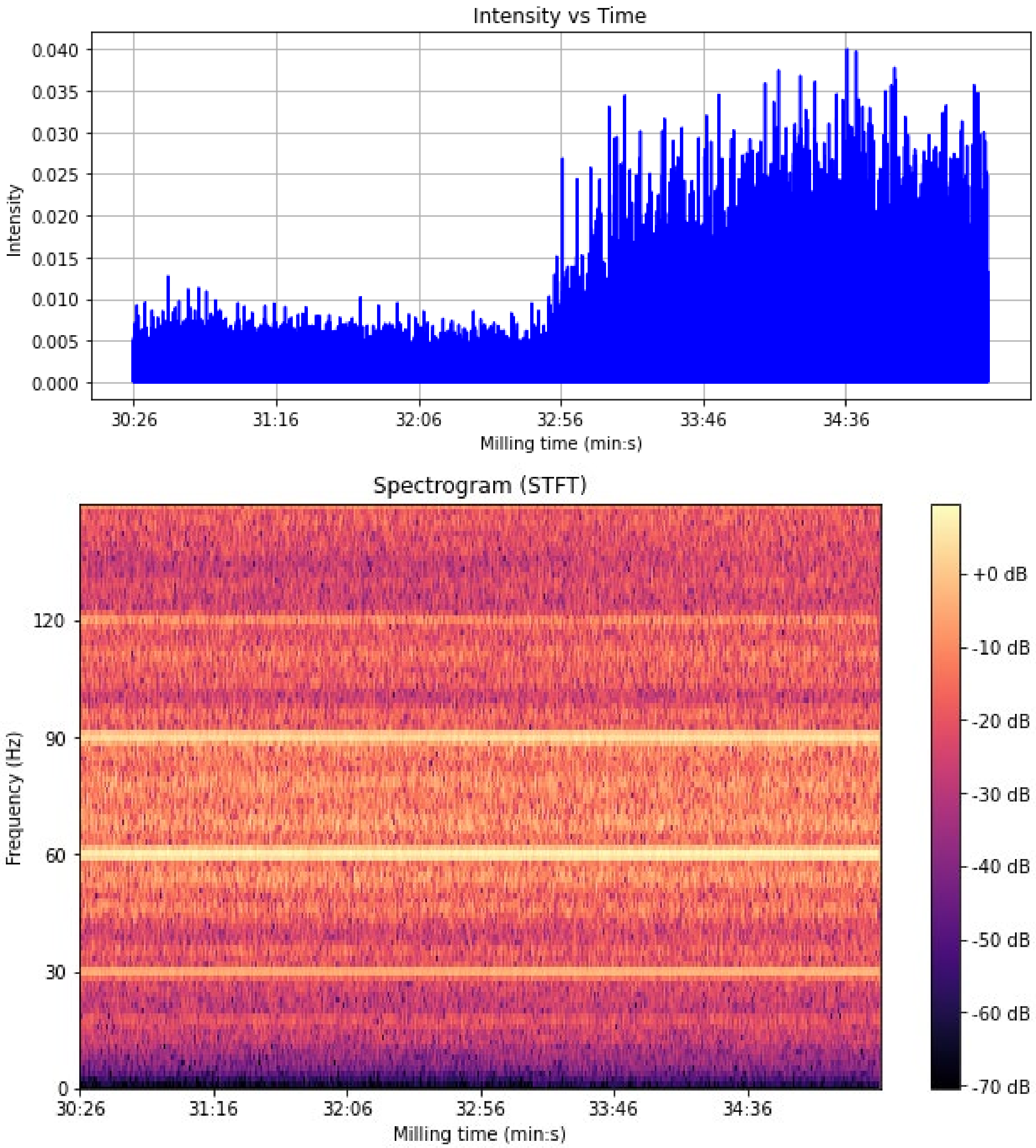

Figure S6f. Intensity spectrum and frequency spectrogram in vicinity of cohesive transition corresponding to formation of a cohesive state under N_2 with 8 x 10 mm spheres and 2.0 g PS (conditions detailed in Table 1 in main text as Exp. 3).

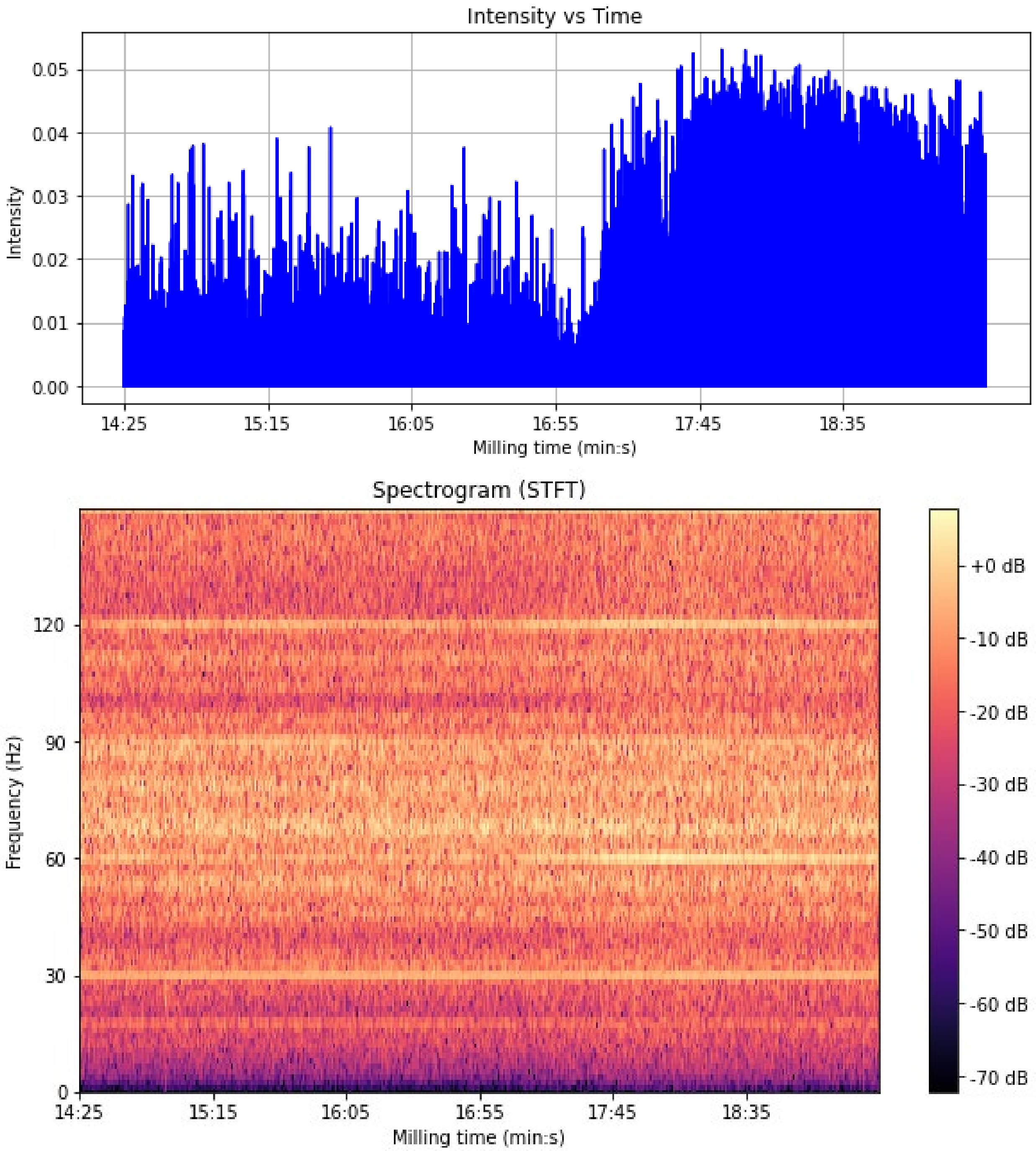

Figure S6g. Intensity spectrum and frequency spectrogram in vicinity of cohesive transition corresponding to the first formation of a cohesive state under air with 8 x 10 mm spheres and 1.0 g PS (conditions detailed in Table 1 in main text as Exp. 5).

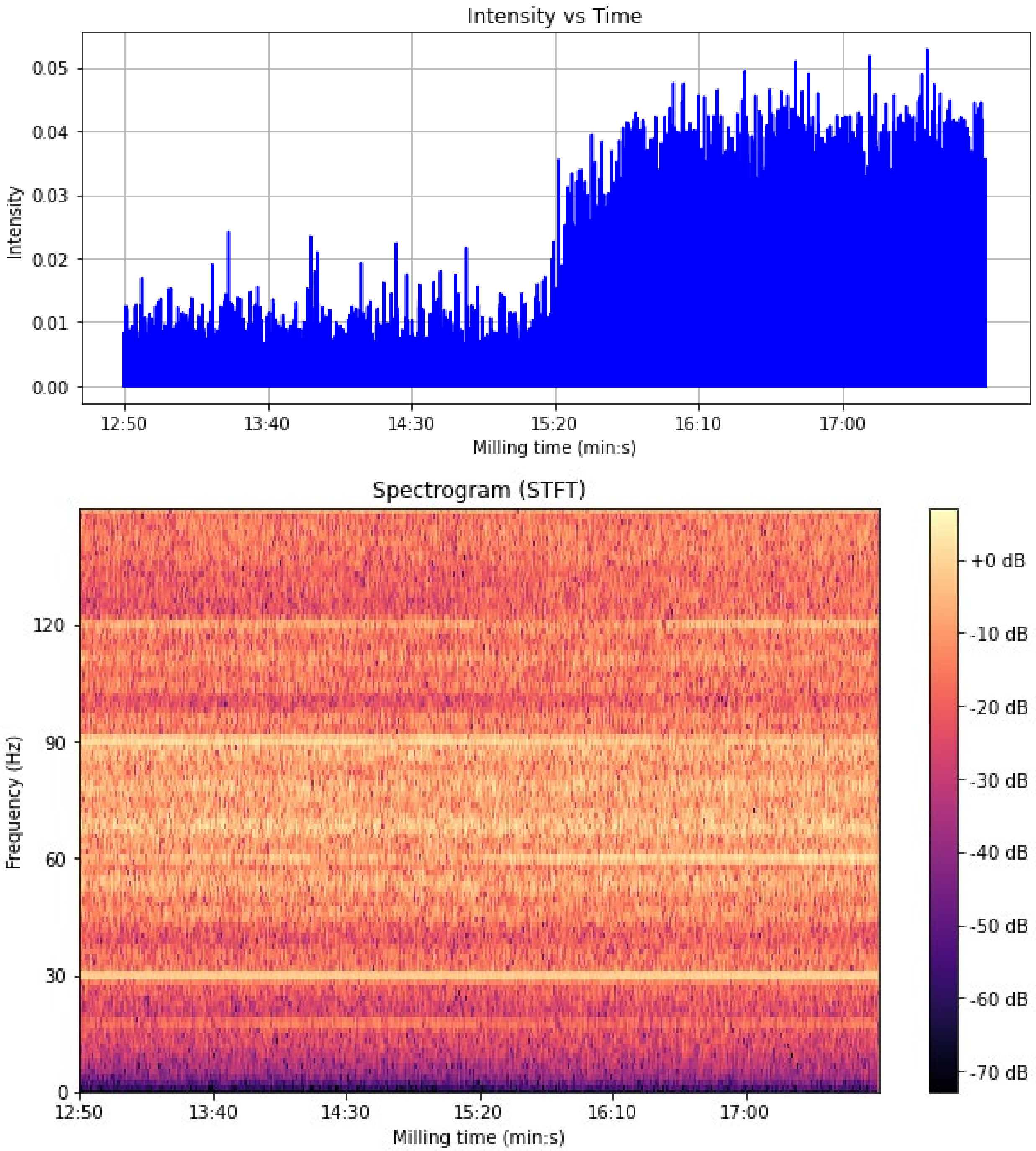

Figure S6h. Intensity spectrum and frequency spectrogram in vicinity of cohesive transition corresponding to second formation of a cohesive state (after pausing milling, cooling reactor down to ambient temperature and restarting milling) under air with 8 x 10 mm spheres and 1.0 g PS (conditions detailed in Table 1 in main text as Exp. 5).

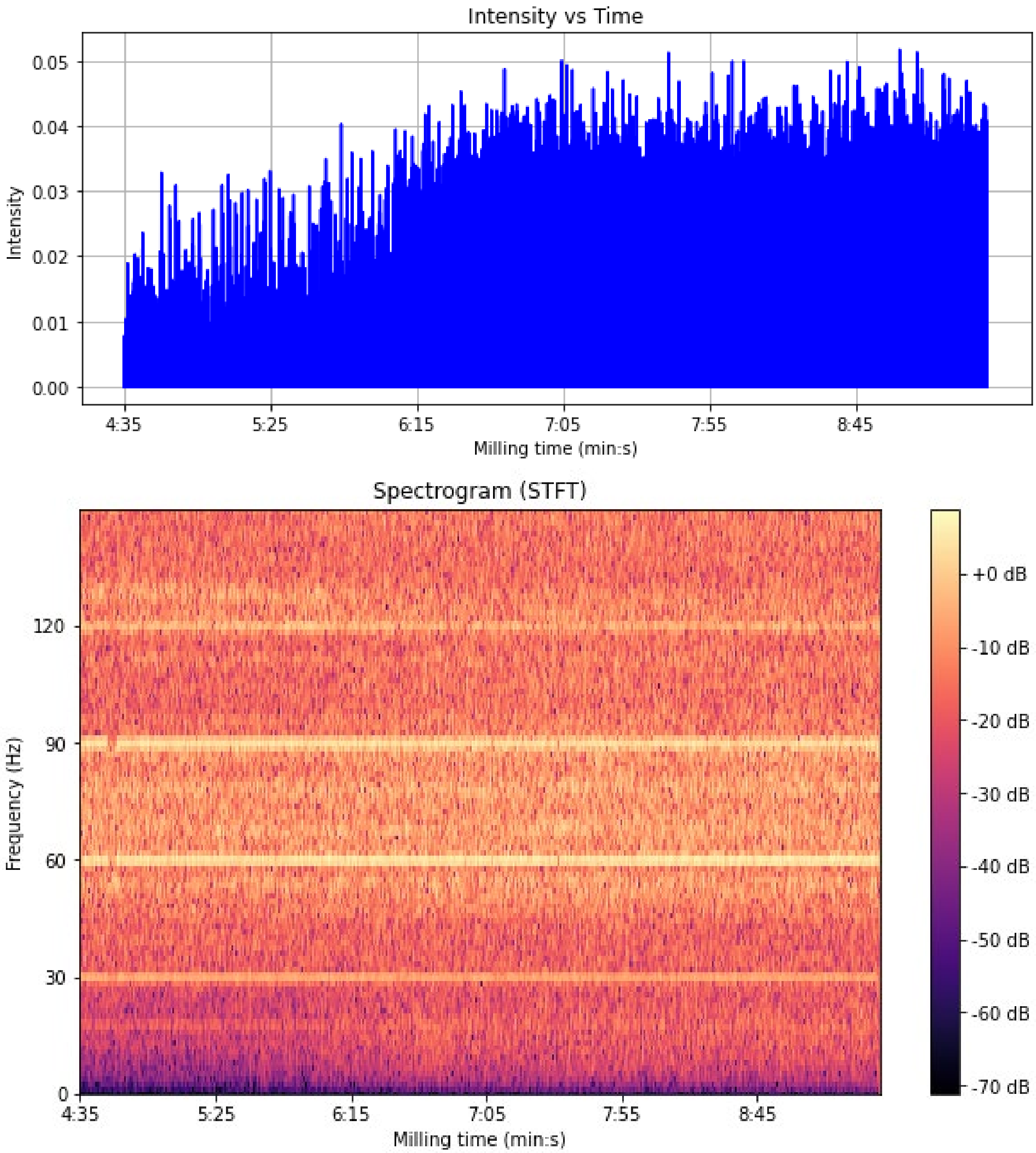

Figure S6i. Intensity spectrum and frequency spectrogram in vicinity of cohesive transition corresponding to formation of a cohesive state under N_2 with 1 x 19 mm sphere and 1.0 g PS (conditions detailed in Table 1 in main text as Exp. 8).

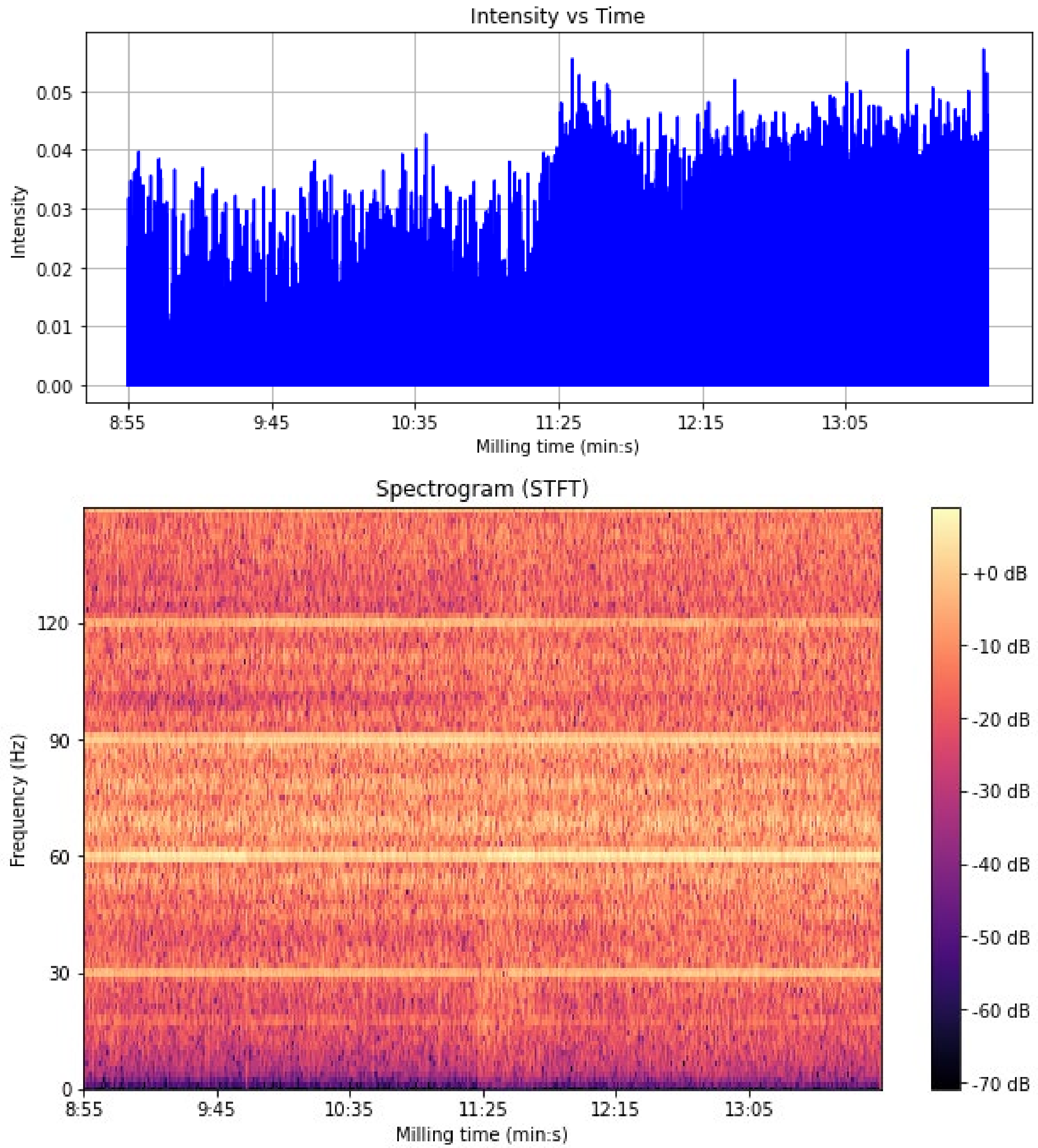

Figure S6j. Intensity spectrum and frequency spectrogram in vicinity of cohesive transition corresponding to formation of a cohesive state under N_2 with 2 x 15 mm spheres and 1.0 g PS (conditions detailed in Table 1 in main text as Exp. 9).

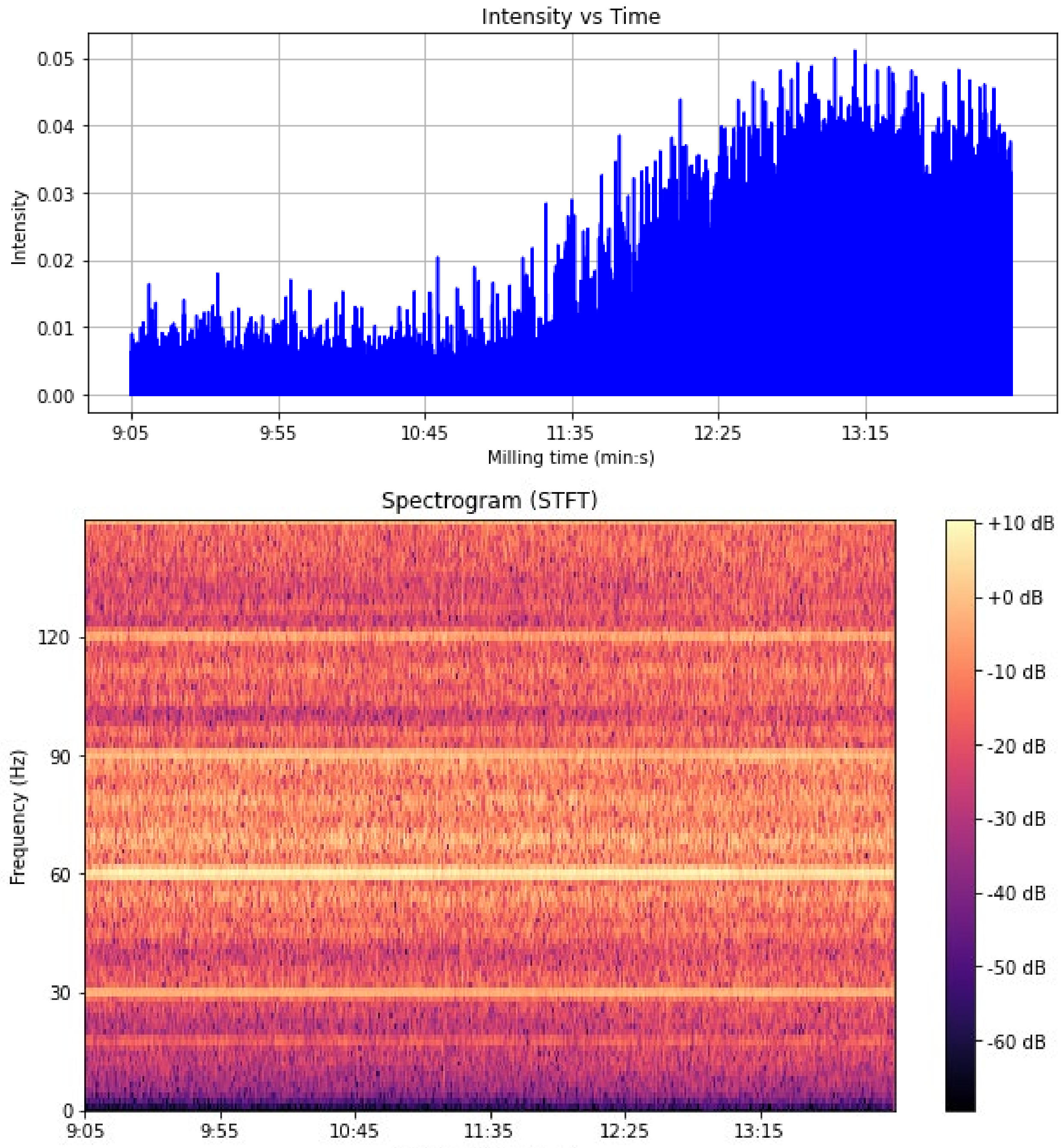

Figure S6k. Intensity spectrum and frequency spectrogram in vicinity of cohesive transition corresponding to formation of a cohesive state under N_2 with 4 x 12 mm spheres and 1.0 g PS (conditions detailed in Table 1 in main text as Exp. 10).

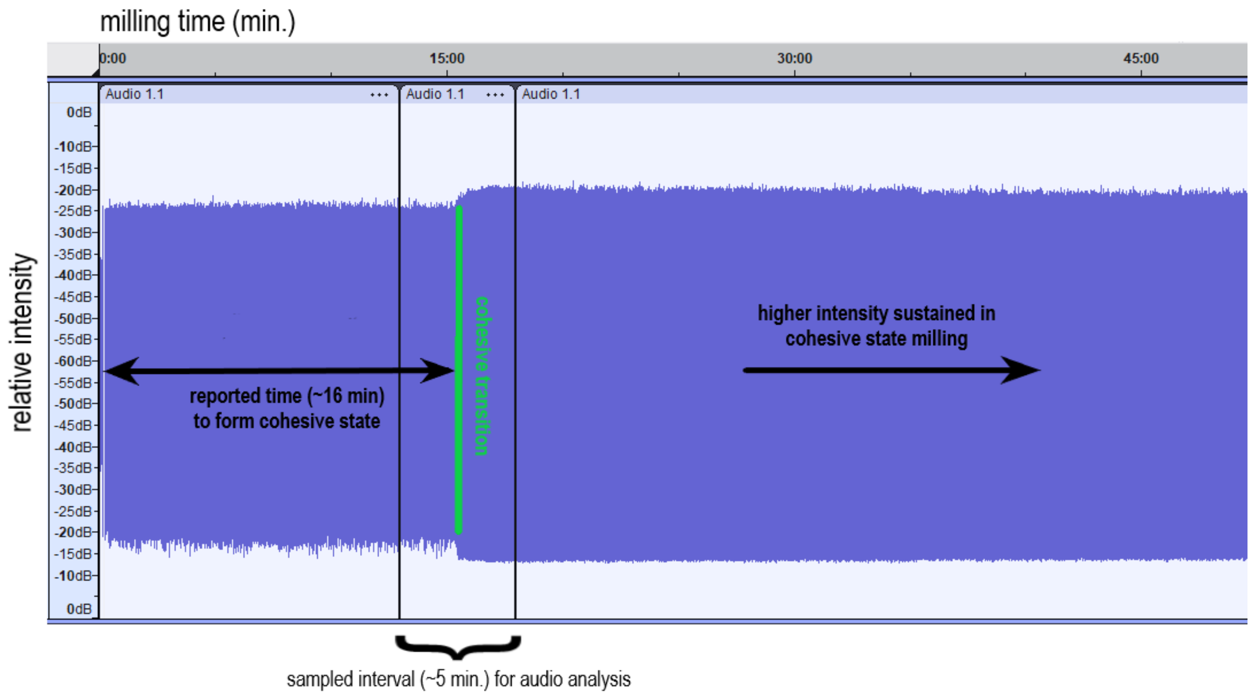

Figure S7. Screenshot of raw audio recording data corresponding to Figure S6c, illustrating how the processed data was clipped from the raw audio. Also indicated is visual identification of the cohesive transition as a sudden increase in intensity range at 16 min. (see Exp. 1, Table 1 in main text) into ball milling.

S.F. Model of Thermodynamically-Limited Poly(styrene) Depolymerization

The following is adapted from Chang[1] and Chang et al.[2]

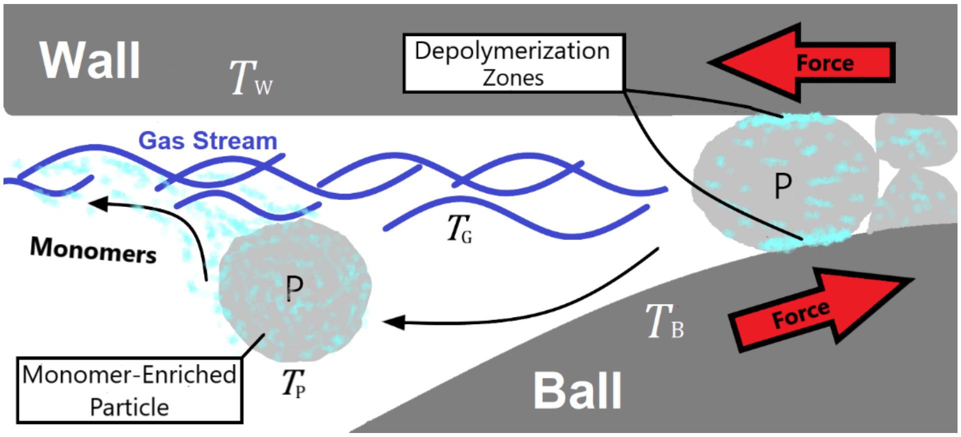

Figure S8. Schematic illustration of basic features of the steady-state mechanochemical PS depolymerization system including relevant variables discussed in the model.

In mechanochemical reactors such as a vibratory ball mill, mechanochemical depolymerization events are created when grinding bodies (reactor wall and balls) collide due to mechanical agitation of the reactor which crush small quantities of solid polymer powder in between their surfaces.[3] A simplified model of the the physical system is illustrated in Figure S8, consisting of macroscopic grinding surfaces divided between the reactor interior wall "W" and grinding spheres "B", and microscopic solid polymer (PS) particles "P", which are associated with average steady state surface temperatures T_W, $T_\mathrm{B} \approx T_\mathrm{W}$, and T_P, respectively. All space that is not occupied by these solid bodies is filled by a constant composition gas phase "G" at temperature T_G and pressure p_0. This gas phase is taken to be pure nitrogen (N_2) to reflect typically reported experimental conditions.[4], [5] When a polymer particle is crushed between two grinding surfaces, mechanochemical processes occur which can lead to the production of monomers (illustrated as light blue blotches in Figure S7) during the course of the impact, with monomers exiting the reactor as vapor in the gas stream.

In cohesive state depolymerization, free flowing PS particles are replaced by a layer of continuous polymer coating over the grinding sphere. This does change the essential characteristics of the model, except that better contact between the grinding sphere and polymer allows for more efficient heat transfer, and we may approximate $T_\mathrm{p} \approx T_\mathrm{B} \approx T_\mathrm{W}$.

For the physical mechanism of a depolymerization event, we adopt an idea proposed by Carta et al.:[6] when the particles are subject to mechanical impact, mechanochemical reactions of solid particles occur predominantly in small pockets of "activated" volumes. Adapting this model to mechanochemical depolymerization, whenever a group of polymer particles is impacted in between two colliding grinding surfaces, a transient spell of depropagation occurs at microscopic regions on these particles where there are chain end radicals. Let $\mathrm{P}_N^{\cdot}$ denote a polymer chain of N monomers which bears a chain end radical. The thermodynamic viability of monomer production can be assessed based on the equilibrium of a single propagation–depropagation reaction step on $\mathrm{P}_N^{\cdot}$ and its associated equilibrium constant K_N:

$$\mathrm{P}_{N-1}^{\cdot} + \mathrm{M} \underset{K_N}{\leftrightarrow} \mathrm{P}_N^{\cdot}\,, \qquad \text{(A1)}$$

where M denotes the monomer species. Thermodynamically, we characterize the depropagation events by a molar Gibbs free energy of *polymerization* $\Delta_r G$ via:

$$\Delta_r G = \Delta_r H - T\Delta_r S, \qquad \text{(A2)}$$

where $\Delta_r H$ and $\Delta_r S$ are the enthalpy and entropy of polymerization respectively, and $T \geq T_\mathrm{B}$ is the temperature at which the transient depolymerization occurs.

The energies and entropy in eqn. A2 are not standard condition values so they are themselves functions of T. However, for simplicity we assume the condition of standard pressure $p^\circ = 101325$ Pa for all thermodynamic functions. To evaluate $\Delta_r G$, we make use of thermodynamic functions relating the molar enthalpy $\Delta_r H$ and the molar entropy $\Delta_r S$ of polymerization to the isobaric heat capacity of the reaction $\Delta_r \bar{C}$:

$$\Delta_r H^\circ + \int_{T^\circ}^{T} (\Delta_r \bar{C}) dT = \Delta_r H(T), \tag{A3}$$

$$\Delta_r S^\circ + \int_{T^\circ}^{T} \left(\frac{\Delta_r \bar{C}}{T}\right) dT = \Delta_r S(T). \tag{A4}$$

By assuming that depropagation reactions convert polymer within a microscopic activated region to gaseous monomers, $\Delta_r \bar{C}$ can be accessed by taking the difference between the isobaric heat capacities of the pure (solid) polymer $\bar{C}_\mathrm{P}$ and the pure monomer $\bar{C}_\mathrm{M}$ in the gas state:

$$\Delta_r \bar{C} = \bar{C}_\mathrm{P} - \bar{C}_\mathrm{M} \tag{A5}$$

$\Delta_r H^\circ$, $\Delta_r S^\circ$, $\bar{C}_\mathrm{P}(T)$ and $\bar{C}_\mathrm{M}(T)$ are available in the literature for PS and its monomer. The sources of thermodynamic data are NIST for monomer styrene, Gaur & Wunderlich[7] for $\bar{C}_\mathrm{P}$ and Dainton & Ivin[8] for $\Delta_r H^\circ$ and $\Delta_r S^\circ$.

The relation between eqn. A2 and K_N associated with eqn. A1 is according to the standard definition of the equilibrium constant, which can also be expressed as a ratio of species activities a:

$$\frac{1}{K_N} = \frac{a_{[\mathrm{P}^\cdot_N]}}{a_{[\mathrm{P}^\cdot_{N-1}]} a_\mathrm{M}} = \exp\left(-\frac{\Delta_r G}{\bar{R}_0 T}\right), \tag{A6}$$

where R is the gas constant, a_M is the monomer activity and the other two activities denote those of the polymer chain end radicals that differ by one monomer unit. Note that K_N is the equilibrium constant with respect to depropagation as the forward reaction, whereas $\Delta_r G$ is the free energy with respect to propagation (as commonly tabulated in literature). For long chains, K_N is practically independent of N.[9] Thus, there should be no difference in activity between reactive chain end radicals belonging to chains of different lengths,[10] so

$$\frac{a_{[\mathrm{P}^\cdot_N]}}{a_{[\mathrm{P}^\cdot_{N-1}]}} \approx 1. \tag{A7}$$

As an example of the applicability of eqn. A7 to our conditions, PS has $N \approx 100 \gg 1$ at its limiting MW of 10,000 g/mol in mechanical degradation.[11] We now apply eqn. A7 to eqn. A6, write K_N as K_0, and equate it to the monomer activity a_M, leading to a simple relationship between a_M and the Gibbs energy of polymerization $\Delta_r G$:

$$K_0 = a_\mathrm{M} = \exp\left(\frac{\Delta_r G}{\bar{R}_0 T}\right). \tag{A8}$$

We may use eqn. A8 directly to plot the depropagation equilibrium constant for PS as a function of T, which can be termed the local temperature at which depropagation occurs during grinding impacts. The result is depicted in Figure S9.

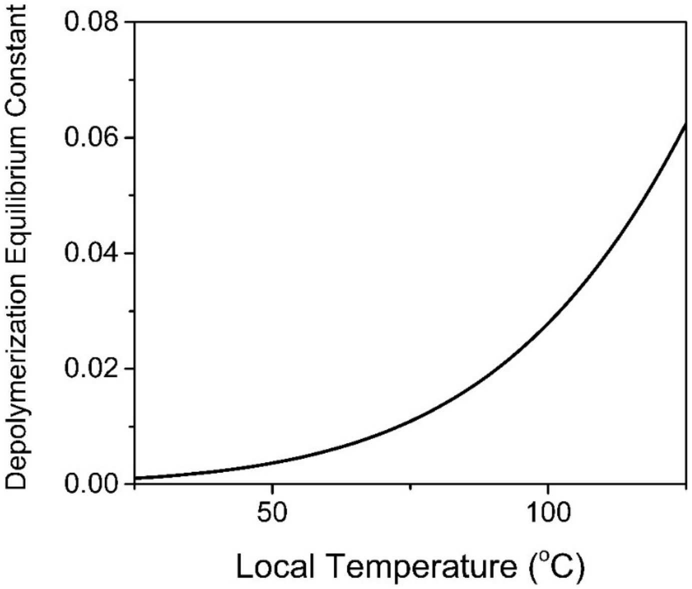

Figure S9. Depolymerization equilibrium constant for PS – equal to monomer activity a_M as a function of local temperature in depolymerization zone.

References

[1] Y. Chang, “The kinetics of ball mill mechanochemistry in its application to the depolymerization of poly(styrene),” Georgia Institute of Technology, 2025.

[2] Y. Chang *et al.*, “Thermodynamic limits of the depolymerization of poly(olefin)s using mechanochemistry,” *RSC Mechanochemistry*, vol. 1, no. 5, pp. 504–513, 2024, doi: 10.1039/D4MR00079J.

[3] A. W. Tricker *et al.*, “Stages and Kinetics of Mechanochemical Depolymerization of Poly(ethylene terephthalate) with Sodium Hydroxide,” *ACS Sustain. Chem. Eng.*, vol. 10, no. 34, pp. 11338–11347, Aug. 2022, doi: 10.1021/acssuschemeng.2c03376.

[4] Y. Chang *et al.*, “Kinetic Phenomena in Mechanochemical Depolymerization of Poly(styrene),” *ACS Sustain. Chem. Eng.*, vol. 12, no. 1, pp. 178–191, Jan. 2024, doi: 10.1021/acssuschemeng.3c05296.

[5] Y. Chang, A. H. Hergesell, C. L. Seitzinger, A. M. Hepstall, I. Vollmer, and C. Sievers, “Stages of Mechanochemical Depolymerization of Poly(styrene) Powder in Oxidative and Inert Atmospheres,” *ACS Sustain. Chem. Eng.*, vol. 13, no. 44, pp. 18970–18982, Nov. 2025, doi: 10.1021/acssuschemeng.5c05942.

[6] M. Carta, E. Colacino, F. Delogu, and A. Porcheddu, “Kinetics of mechanochemical transformations,” *Phys. Chem. Chem. Phys.*, vol. 22, no. 26, pp. 14489–14502, Jul. 2020, doi: 10.1039/D0CP01658F.

[7] U. Gaur and B. Wunderlich, “Heat Capacity and Other Thermodynamic Properties of Linear Macromolecules. V. Polystyrene,” *J. Phys. Chem. Ref. Data*, vol. 11, no. 2, pp. 313–325, Apr. 1982, doi: 10.1063/1.555663.

[8] F. S. Dainton and K. J. Ivin, “Changes of entropy and heat content during polymerization,” *Trans. Faraday Soc.*, vol. 46, p. 331, 1950, doi: 10.1039/tf9504600331.

[9] J. Sohma, “Mechanochemistry of polymers,” *Prog. Polym. Sci.*, vol. 14, no. 4, pp. 451–596, Jan. 1989, doi: 10.1016/0079-6700(89)90004-X.

[10] F. S. Dainton and K. J. Ivin, “Reversibility of the Propagation Reaction in Polymerization Processes and its Manifestation in the Phenomenon of a ‘Ceiling Temperature’’,’” *Nature*, vol. 162, no. 4122, pp. 705–707, Oct. 1948, doi: 10.1038/162705a0.

[11] H. Staudinger and W. Heuer, “Über hochpolymere Verbindungen, 93. Mitteil.: Über das Zerreißen der Faden-Moleküle des Poly-styrols,” *Berichte der Dtsch. Chem. Gesellschaft (A B Ser.*, vol. 67, no. 7, pp. 1159–1164, Jul. 1934, doi: 10.1002/cber.19340670708.
